# Bioinformatics identification and transcript profile analysis of the mitogen-activated protein kinase gene family in the diploid woodland strawberry *Fragaria vesca*

**DOI:** 10.1371/journal.pone.0178596

**Published:** 2017-05-31

**Authors:** Wei Wei, Zhuangzhuang Chai, Yinge Xie, Kuan Gao, Mengyuan Cui, Ying Jiang, Jiayue Feng

**Affiliations:** 1 State Key Laboratory of Crop Stress Biology for Arid Areas, College of Horticulture, Northwest A&F University, Yangling, Shaanxi, China; 2 Key Laboratory of Protected Horticulture Engineering in Northwest China, Ministry of Agriculture, Yangling, Shaanxi, China; Hainan University, CHINA

## Abstract

Mitogen-activated protein kinases (MAPKs) play essential roles in mediating biotic and abiotic stress responses in plants. However, the *MAPK* gene family in strawberry has not been systematically characterized. Here, we performed a genome-wide survey and identified 12 *MAPK* genes in the *Fragaria vesca* genome. Protein domain analysis indicated that all FvMAPKs have typical protein kinase domains. Sequence alignments and phylogenetic analysis classified the *FvMAPK* genes into four different groups. Conserved motif and exon-intron organization supported the evolutionary relationships inferred from the phylogenetic analysis. Analysis of the stress-related *cis*-regulatory element in the promoters and subcellular localization predictions of *FvMAPKs* were also performed. Gene transcript profile analysis showed that the majority of the *FvMAPK* genes were ubiquitously transcribed in strawberry leaves after *Podosphaera aphanis* inoculation and after treatment with cold, heat, drought, salt and the exogenous hormones abscisic acid, ethephon, methyl jasmonate, and salicylic acid. RT-qPCR showed that six selected *FvMAPK* genes comprehensively responded to various stimuli. Additionally, interaction networks revealed that the crucial signaling transduction controlled by FvMAPKs may be involved in the biotic and abiotic stress responses. Our results may provide useful information for future research on the function of the *MAPK* gene family and the genetic improvement of strawberry resistance to environmental stresses.

## Introduction

Strawberry (*Fragaria × ananassa* Duch.) is nutritiously and economically one of the most important fruits crops grown worldwide for the production of fresh fruit and juice. However, strawberry growth and production are hindered by multiple biotic and abiotic stresses, such as pathogen infection, limited water availability, soil salinization and extreme temperatures [1, 2]. Therefore, systematic identification and functional study of stress response and tolerance genes in strawberry are required to elucidate the molecular mechanisms of strawberry tolerance and susceptibility. To regulate their development and cope with environmental stress, plants have developed sophisticated mechanisms to perceive external signals and respond to them with appropriate physiological and morphological alterations [3]. Protein phosphorylation via protein kinases is one of the major mechanisms controlling the intracellular response to extracellular signals [4]. Mitogen-activated protein kinases (MAPKs), which are widely distributed in eukaryotes, are a specific class of serine/threonine protein kinases that transduce extracellular signals into intracellular responses through phosphorylation [5]. The MAPK signaling cascade in plants typically consists of functionally interlinked protein kinases: MAP kinase kinase kinase (MAPKKK, MAP3K), MAP kinase kinase (MAPKK, MKK) and MAP kinase (MAPK, MPK) [6].

MAPK contains eleven domains (I–XI), and the highly conserved threonine and tyrosine residues (TXY motif or T-loop) existing between domains VII and VIII form the activation loop, which is believed to undergo phosphorylation to activate MAPKs [6, 7]. MAPKs can be divided into four major groups (A, B, C, and D) based on the presence of the TEY or TDY motif in their phosphorylation sites [6]. MAPKs belonging to groups A, B, and C possess a Thr-Glu-Tyr (TEY) motif in their activation loop, while members of group D harbor a Thr-Asp-Tyr (TDY) motif in their activation loop and an extended C-terminal region [6].

Accumulating evidence suggests that plant *MAPKs* are involved in the regulation of biotic and abiotic stresses, including pathogen infection, heat, cold, drought and salt stress [8–11], as well as in the production of their related hormonal signals, such as abscisic acid (ABA), salicylic acid (SA), jasmonic acid (JA), and ethylene (Eth) [12, 13]. In plants, the most extensively studied *MAPKs*, i.e., *MPK3*, *MPK4* and *MPK6* in *Arabidopsis* and their orthologs in other plant species, are mainly involved in stress responses. For example, MEKK1–MKK4/5–MPK3/6–WRKY22/WRKY29 plays an important role in plant innate immunity [14]. The MEKK1–MKK1/MKK2–MPK4–MKS1/WRKY33 cascade negatively controls plant immune responses [15–17]. The MEKK1-MKK2-MAPK4/6 cascade was shown to be activated by cold and salt stress [18]. Recent research has shown that the MAPKKK5-MKK4/5-MPK3/6 pathway plays an important role in chitin signaling in *Arabidopsis* [19]. *AtMPK3* and *AtMPK6* are also involved in various abiotic stress response and hormone signaling pathways [10, 20–22]. *MAPK* genes in several important crops have also attracted considerable attention. For example, *OsMPK5* is induced by ABA as well as various biotic and abiotic stresses [23], and *ZmMPK17*, a novel maize group D MAP kinase gene, is involved in multiple stress responses [24]. Additionally, several *MAPK* genes have been found to play an important role in the regulation of developmental processes [25].

The availability of complete genome sequences has allowed genome-wide surveys of *MAPK* genes in plants such as *Arabidopsis thaliana* [6], rice (*Oryza sativa*) [26], tomato (*Solanum lycopersicum*) [27], maize (*Zea mays*) [28], apple (*Malus domestica*) [29], grape (*Vitis vinifera*) [30], watermelon (*Citrullus lanatus*) [31], and cassava (*Manihot esculenta*) [32]; these surveys indicated that several *MAPK* genes are transcribed at high levels under various stresses. The sequenced diploid woodland strawberry *Fragaria vesca* accession Hawaii-4 with a relatively small genome (240 Mb, 2n = 2x = 14) provides a good platform for our understanding of a genome-wide analysis of *MAPK* genes [33, 34]. ‘Heilongjiang-3’ strawberry, which is naturalized in the Heilongjiang province of China, was identified as the wild diploid woodland strawberry *F*. *vesca*. Previous research on the *MAPK* gene family in *Fragaria vesca* identified 11 members [35]. However, a prior study only focused on conserved domains and the activation loop without transcript profile analysis. Here, we identified 12 *MAPKs* in the diploid woodland strawberry (*F*. *vesca*) accession Heilongjiang-3 and analyzed their phylogenetic relationship, protein domain, exon-intron organizations, conserved motif, *cis*-acting elements, subcellular localization, and interaction networks. Further, we also characterized the transcript patterns of *FvMAPK* genes in diverse tissues and in the response to stresses and hormone treatment. The current results may provide a novel insight into future work on the function of strawberry *MAPK* genes in response to biotic and abiotic stresses.

## Materials and methods

### Genome-wide identification of *MAPK* genes in strawberry

To identify the *MAPK* genes in the strawberry genome (*F*. *vesca*), two database searches were performed. Sequences of 20 *Arabidopsis* MAPK proteins were retrieved from the *Arabidopsis* Information Resource (TAIR, https://www.arabidopsis.org/) and used as queries to perform BLAST-P searches against the National Center for Biotechnology Information database (*Fragaria vesca* Annotation Release 101) (NCBI, http://www.ncbi.nlm.nih.gov). Additionally, all protein sequences were then used as queries to perform BLAST-P searches against the Strawberry Genome Database (https://www.rosaceae.org/) [6]. Only hits with e-values lower than 1e^-10^ were retrieved as candidates. Self-BLAST of the obtained sequences against the NCBI database was carried out to remove redundancies. The Pfam database (http://pfam.xfam.org/search/) and the Conserved Domain Database at NCBI (CDD, http://www.ncbi.nlm.nih.gov/Structure/cdd/wrpsb.cgi/) were used to confirm that all putative non-redundant sequences contained the canonical consensus sequences for serine/threonine protein kinases and the essential TDY or TEY signature motif in the activation loop. Additionally, all putative candidates were manually verified using the InterProScan program (http://www.ebi.ac.uk/Tools/pfa/iprscan/) to confirm they contained the MAP kinase domain (IPR003527), protein kinase domain (IPR000719), ATP-binding site (IPR017441) and serine/threonine protein kinase active site (IPR008271). The parameters of the deduced strawberry MAPK proteins, such as isoelectric points and molecular weights, were calculated using the ExPASy Proteomics Server (http://web.expasy.org/compute_pi/).

### Bioinformatics analysis of *MAPK* genes in strawberry

Multiple sequence alignment was performed using ClustalX 2.0.12 with the default settings for strawberry MAPK protein sequences, and illustrations were produced using GeneDoc software. The full-length amino acid sequences of the MAPK proteins from strawberry (FvMAPK), *A*. *thaliana* (AtMAPK), apple (MdMAPK), rice (OsMAPK), tomato (SlMAPK), maize (ZmMAPK), and grape (VvMAPK) were retrieved from previous reports and used for phylogenetic tree construction. The phylogenetic tree for the MAPK proteins was constructed using the neighbor-joining (NJ) method in the MEGA 6.0 program. Neighbor-joining analysis with pairwise deletion and bootstrap analysis with 1000 replicates were performed using the p-distance model [36]. Exon-intron organization of strawberry *MAPKs* was illustrated using the Gene Structure Display Serve 2.0 (GSDS, http://gsds.cbi.pku.edu.cn/) by comparison of coding sequence with their corresponding genomic sequences. The upstream 1-kb region of the translation start site of the *FvMAPK* genes was used for putative promoter *cis*-acting element analysis in PlantCARE (http://bioinformatics.psb.ugent.be/webtools/plantcare/html/), and the analysis results were manually drawn using PowerPoint 2016. The conserved domain annotation was performed using the InterProScan program (http://www.ebi.ac.uk/Tools/pfa/iprscan/). The conserved motifs of the FvMAPK proteins were obtained from online Multiple Expectation Maximization for Motif Elicitation (MEME, http://meme-suite.org/tools/meme) using the following parameters: minimum motif width, 6; maximum motif width, 100; and maximum number of motifs, 13. Subcellular localization predictions of each FvMAPK were performed using WoLF PSORT (http://www.genscript.com/wolf-psort.html) with the organism set as ‘Plant’, CELLO v2.5 (http://cello.life.nctu.edu.tw/) with the organism set as ‘Eukaryotes’, Plant-PLoc (http://www.csbio.sjtu.edu.cn/bioinf/plant/), and ProtComp 9.0 (http://linux1.softberry.com/berry.phtml?topic=protcomppl&group=programs&subgroup=proloc). A specific interaction network with experimental evidence of MPK3, MPK4 and MPK6 was constructed using the online program STRING 10 (http://string-db.org/) with the organism specified as “*Arabidopsis thaliana*” and option value >0.900. Then, the homologs of these interactive proteins in strawberry were identified using reciprocal best BLAST-P analysis.

### Plant materials and treatments

The wild diploid strawberry *F*. *vesca* accession Heilongjiang-3 was used in this study. The plants were grown at a 22/18°C day/night temperature and a relative humidity of 75% without supplemental light in the strawberry germplasm resource greenhouse at Northwest A&F University in China. Strawberry organs and tissues, including roots, stems, runners, young leaves, mature leaves, flowers, and fruits, were collected and stored at –80°C for tissue-specific analysis. Strawberry seedlings aged six months, by which time the tenth leaf was fully expanded, were selected for the stress treatments. Pathogen treatment was carried out by inoculating the young leaves of ‘Heilongjiang-3’ with powdery mildew (*Podosphaera aphanis*) as previously described with minor modifications [36]. Leaves were harvested at 0, 24, 48, 72, 96, 120, 144, and 168 h post-inoculation (hpi) (uninoculated leaves were used as a negative control). For the salt treatments, potted strawberry plants were irrigated with 300 mM NaCl (distilled water was used as a mock control). For the heat and cold treatments, the plantlets were placed in a growth chamber at a high temperature (42°C) or a low temperature (4°C; range from 22 to 27°C was used as a mock control). For the hormone treatments, strawberry leaves were sprayed with 0.1 mM abscisic acid (ABA), 1 mM salicylic acid (SA), 0.1 mM methyl jasmonate (MeJA), or 0.5 g/L ethephon (Eth) (distilled water was used as a solvent control). The leaves of strawberry plants subjected to the salt, heat, cold, and hormone treatments were harvested at 0, 0.5, 2, 4, 8, 12, 24, and 48 h post-treatment (hpt). For the drought treatment, the strawberry plants were treated according to the methods of a previous study [36]. At each time point of each treatment, six leaves from six separate plants were combined to form one sample, and all treatments were evaluated in triplicate. All collected plant samples were immediately frozen in liquid nitrogen and stored at –80°C until use.

### Gene transcript analysis

Total RNA was extracted from treated leaves or tissue samples using an EZNA Plant RNA Kit (R6827-01, Omega Bio-tek, USA) according to the manufacturer’s instructions. RNA integrity was verified by 1.2% agar gel electrophoresis, and the RNA concentration was measured using a NanoDrop 1000 (Thermo, USA). The PrimeScript™ RT reagent Kit with gDNA Eraser (TaKaRa Biotechnology, Dalian, China) was used to remove any genomic DNA contamination, and first-strand cDNA was synthesized following the manufacturer’s protocol. Approximately 1.5 μg of RNA was used for each 20-μL reaction. The concentration of cDNA was adjusted according to the interspacer 26S-18S strawberry RNA housekeeping gene *FvRib413*. We also used an additional internal reference gene, *FvGAPDH2*, to evaluate the quality and consistency of the cDNA [37, 38]. Gene-specific primers (S1 Table) were designed for each *FvMAPK* gene using VECTOR NTI. Semi-quantitative reverse-transcription (RT) PCR and reverse transcription quantitative PCR (RT-qPCR) were performed as previously described [39] with some modifications.

### Statistical analysis

All data were statistically analyzed using a paired Student’s t test (http://www.physics.csbsju.edu/stats/). The mean values ± standard deviation of the mean (SD) were calculated from the results of at least three replicates, and significant differences relative to controls are presented at *p < 0.05 and **p < 0.01.

## Results

### Identification and annotation of strawberry *MAPK* genes

To identify *MAPK* genes in *F*. *vesca*, MAPK protein sequences from *Arabidopsis* were used as queries in a BLAST-P search of the publically available NCBI database (*Fragaria vesca* Annotation Release 101) and the Strawberry Genome Database. The redundant candidate sequences or sequences without relevant domains or conserved motifs of the *MAPK* family proteins were excluded from further analysis. After multiple steps of screening and validation of the conserved domains, a total of 12 genes were defined as strawberry *MAPK* genes (Table 1). The newly identified *FvMAPK* genes were designated based on the names of their presumptive *Arabidopsis* orthologs. If two or more strawberry genes had the same homolog in *Arabidopsis*, they were distinguished by an extra number. For example, gene19238 and gene31827 are the homologs of *AtMPK4*; therefore, they were named *FvMAPK4-1* and *FvMAPK4-2*, respectively. Previous work identified 11 *MAPK* genes in strawberry [35]. On this basis, we added one member (*FvMAPK17*, gene04076). Detailed characteristics of the *MAPK* genes in *F*. *vesca* are provided in Table 1.

**Table 1:** Information on the *MAPK* gene family in strawberry.

Name	Gene ID^a^	Accession no.^b^	Length (bp)	No. of aa	Mw^c^ (kDa)	pI^c^	Gene location	Strand	Group
*FvMAPK1*	gene15192	XP_004291845.1	1820	372	42.64	6.09	LG2: 32254545–32257169	-	C
*FvMAPK3*	gene25390	XP_004294685.1	1811	371	42.71	5.62	LG3: 19690549–19693371	-	A
*FvMAPK4*-1	gene19238	XP_004306748.1	1584	373	42.67	6.08	LG7: 4574723–4578939	+	B
*FvMAPK4*-2	gene31827	XP_004298829.1	1805	377	43.24	6.40	LG5: 1784699–1788845	-	B
*FvMAPK6*	gene10128	XP_004287157.1	1695	391	44.71	5.65	LG1: 1081422–1084925	+	A
*FvMAPK7*	gene14943	XP_004287861.1	1879	370	42.64	8.32	LG1: 9041132–9043978	-	C
*FvMAPK9*	gene09401	XP_004301256.2	2249	691	78.45	7.86	LG5: 10188960–10193438	+	D
*FvMAPK13*	gene25407	XP_004294692.1	1852	365	41.85	5.05	LG3: 19870360–19873988	+	B
*FvMAPK16*	gene06108	XP_004296956.1	2804	561	63.83	8.73	LG4: 13067191–13073043	+	D
*FvMAPK17*	gene04076	XP_004297369.2	2237	580	66.33	7.07	LG4: 18603202–18607991	-	D
*FvMAPK19*	gene27365	XP_004294176.1	2008	609	69.37	9.26	LG3: 11156420–11160717	-	D
*FvMAPK20*	gene28706	XP_004303866.1	3272	618	70.68	9.23	LG6: 26819611–26825467	-	D

^a^ Gene IDs are available in the Genome Database for Rosaceae (GDR, https://www.rosaceae.org/).

^b^ Accession numbers are available in the National Center for Biotechnology Information database (*Fragaria vesca* Annotation Release 101) (NCBI, http://www.ncbi.nlm.nih.gov).

^c^ Mw, molecular weight; pI, isoelectric point. Detailed FvMAPK protein characteristics were predicted using the ExPASy online service (http://web.expasy.org/cgi-bin/compute_pi/pi_tool).

### Phylogenetic analysis of *MAPK* genes from strawberry, *Arabidopsis*, apple, grape, maize, rice, and tomato

**Fig 1:**
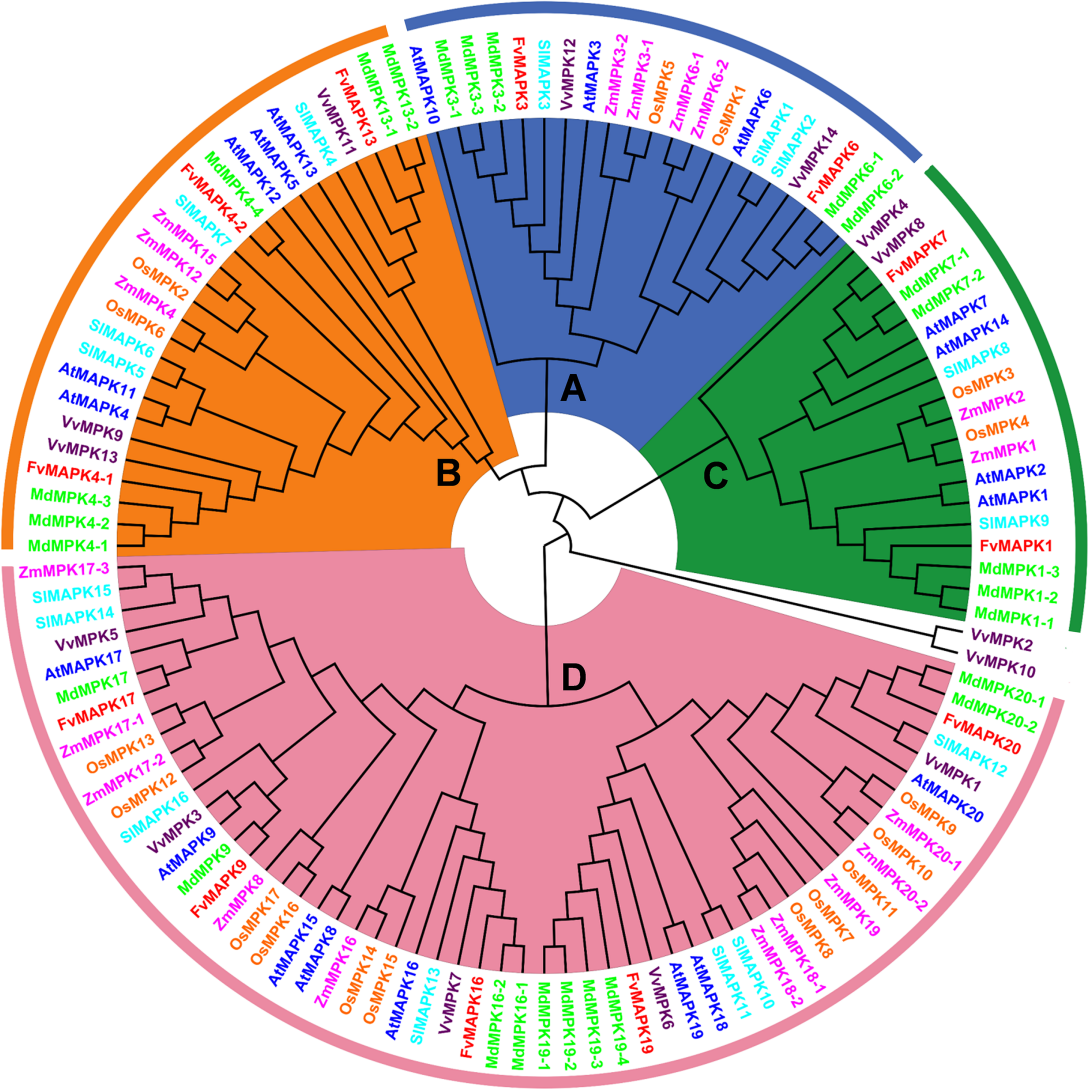
Phylogenetic analysis of *MAPKs* from strawberry, *Arabidopsis*, apple, grape, maize, rice, and tomato. The neighbor-joining tree was created using the MEGA6.0 program with the p-distance model, using full-length sequences of 12 strawberry (Fv), 20 *Arabidopsis* (At), 26 apple (Md), 14 grape (Vv), 19 maize (Zm), 17 rice (Os), and 16 tomato (Sl) MAPK proteins. The bootstrap value was set to 1000 replicates. FvMAPKs are highlighted in red, and the other MAPKs are shown in different colors.

To investigate the classification and evolution and to gain some insight into the potential functions of FvMAPK proteins from well-studied *MAPKs* in other plant species, a total of 124 *MAPK* genes were used to construct a phylogenetic tree, i.e., 12 *FvMAPKs* from strawberry, 20 *AtMAPKs* from *Arabidopsis* [6], 26 *MdMAPKs* from apple [29], 14 *VvMAPKs* from grape [30], 19 *ZmMAPKs* from maize [28], 17 *OsMAPKs* from rice [26], and 16 *SlMAPKs* from tomato [27]. As shown in Fig 1, 12 *FvMAPKs* were classified into four different groups (groups A, B, C, and D) together with their MAPK orthologs in various species. Group A contained two genes (*FvMAPK3*, *FvMAPK6*), group B contained three genes (*FvMAPK4-1*, *FvMAPK4-2*, *FvMAPK13*), and group C contained two genes (*FvMAPK1*, *FvMAPK7*), while group D was the largest clade with five *FvMAPK* genes (*FvMAPK9*, *FvMAPK16*, *FvMAPK17*, *FvMAPK19*, *FvMAPK20*). The classification of *MAPKs* suggests that *FvMAPKs* in different groups might have different functions.

### Multiple sequence alignment and domain analysis of FvMAPK proteins

**Fig 2:**
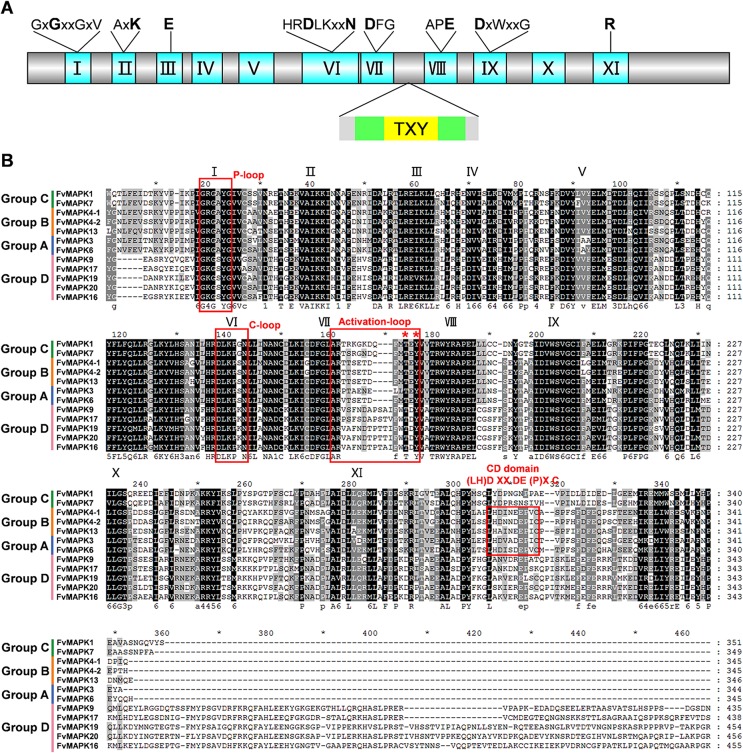
Multiple sequence alignment of the kinase domains of strawberry MAPK proteins. **(A)** Eukaryotic MAPK kinase primary structure: blue areas represent regions containing the eleven domains (I–XI) that are found in all serine/threonine protein kinases; gray areas indicate regions that are less conserved. **(B)** Alignment of the kinase domains for 12 strawberry MAPK proteins. Alignment was performed using ClustalX 2.0.12 and displayed using GeneDoc. Identical sequences are highlighted in black, and similar residues are shown in gray shading. The 11 kinase domains are highlighted using Roman numerals (I to XI) above the sequence. The P-loop, C-loop, activation-loop motifs and CD domain are indicated by red boxes above the alignments. The conserved threonine and tyrosine residues TXY are indicated using asterisks. Fv: *F*. *vesca*.

In previous studies, MAPKs from several plant species were characterized based on the presence of characteristic features of serine/threonine protein kinases, namely, the 11 conserved MAPK domains and the phosphorylation-activation TXY motifs [7]. Sequence alignment indicated that all of the FvMAPK proteins contained highly conserved regions, spanning approximately 300 amino acids near the N-terminal portion, that were composed of 11 characteristic domains (I–XI) (Fig 2). Several highly conserved characteristic subdomains, such as the activation-loop, P-loop and C-loop, were also identified in the FvMAPK proteins (Fig 2). The activation-loop subdomain was present between domains VII and VIII, and the TXY motif, which was phosphorylated for activity, was present in all FvMAPKs (Fig 2). Members in groups A, B and C contained the TEY motif, whereas FvMAPKs in group D had the TDY motif (Fig 2). However, no other TXY variant has been found among all FvMAPKs [35]. In addition, a conserved CD domain with the sequence (LH)DXXDE(P)XC (where X represents any amino acid) was present in groups A and B FvMAPKs but was absent in groups C and D FvMAPKs. The TDY-containing FvMAPKs exhibit extended C-terminal regions, which are generally present in the TDY class of MAPKs from other plants (Fig 2).

**Fig 3:**
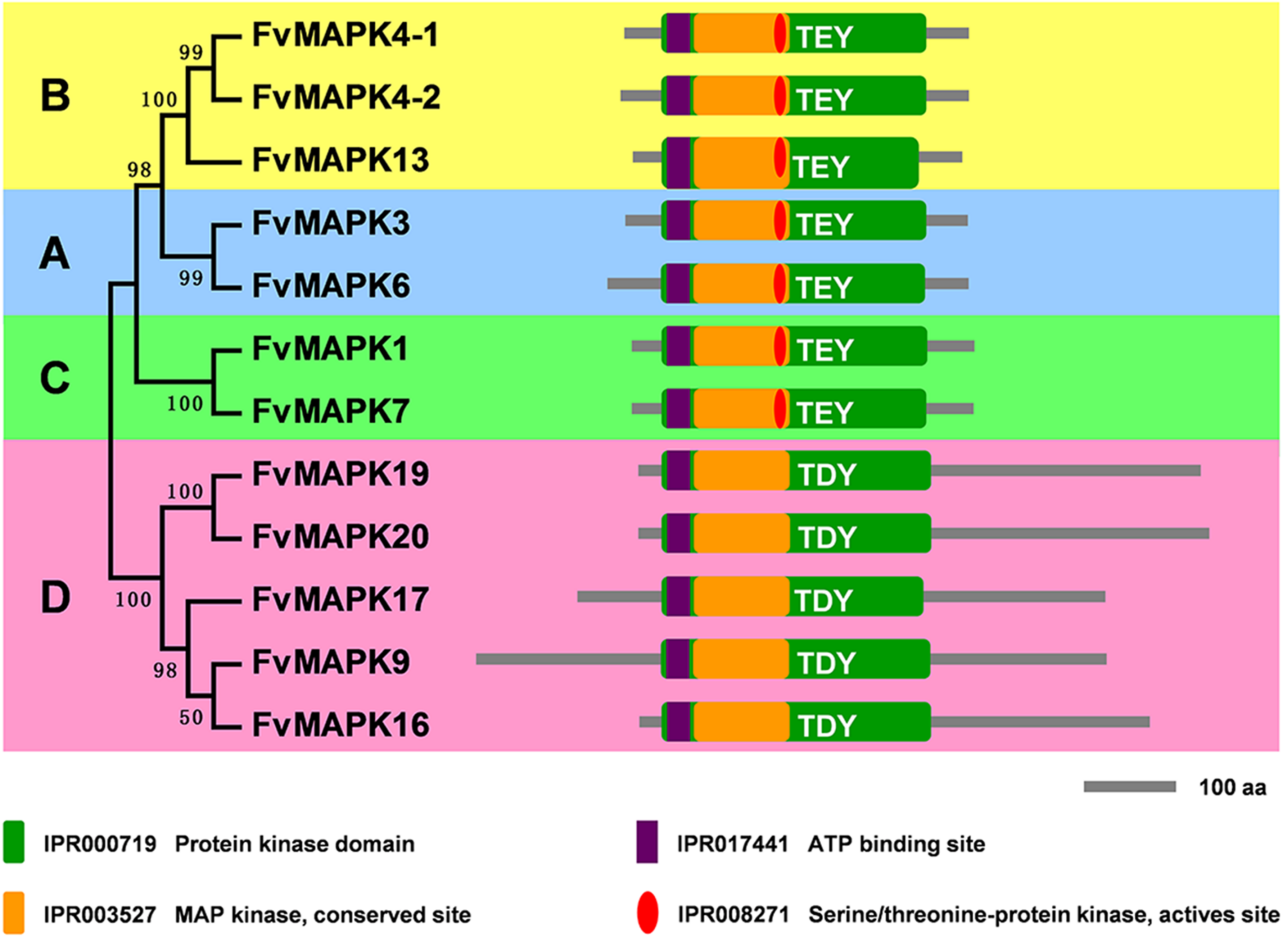
Phylogenetic analysis and domain organization of strawberry MAPKs. The unrooted phylogenetic tree was generated using the MEGA6.0 program with the neighbor-joining method. Bootstrap supports from 1000 replicates are indicated at each branch. The domain organizations were analyzed by scanning the protein sequences for the conserved domains using the InterProScan program.

We predicted the protein domain of the FvMAPKs using InterProScan databases [40]. As shown in Fig 3, each group of the FvMAPK proteins shared a similar protein domain organization based on phylogenetic analysis. All FvMAPKs contained a protein kinase domain (IPR000719) and a MAP kinase domain (IPR003527). In addition, all FvMAPKs were characterized by an ATP-binding site (IPR017441) in the N-terminal extremity of the protein kinase domain. Furthermore, almost all FvMAPK proteins contained the serine/threonine protein kinase active site (IPR008271) in the central part of the protein kinase domain, excluding group D in the FvMAPKs.

### Conserved motif identification and gene structure analysis of *FvMAPK* genes

**Fig 4:**
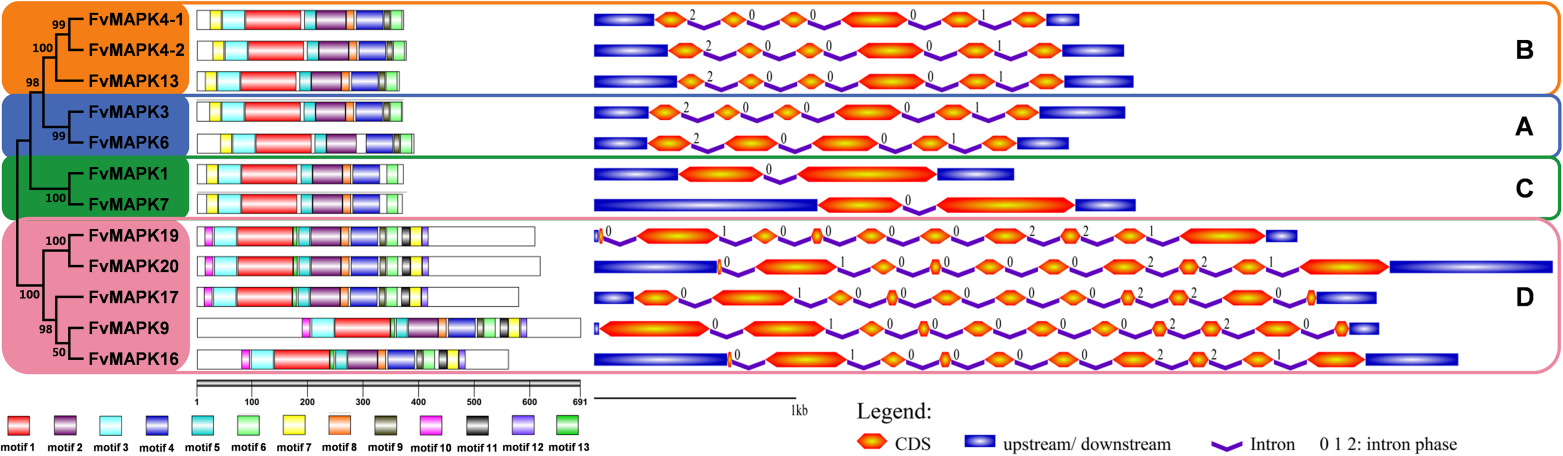
Structural analysis of strawberry *MAPK* genes. Conserved motif of the strawberry *MAPK* genes are shown on the left and are denoted by rectangles with different colors. Exon-intron organization of the strawberry *MAPK* genes are shown on the right, with exons and introns represented by orange double-sided wedges and purple lines, respectively; untranslated regions (UTRs) are indicated using blue rectangles.

To obtain a better understanding of the diversification and evolutionary relationships of the MAPK proteins in *F*. *vesca*, the conserved motifs and exon-intron organization of the *FvMAPKs* were analyzed. The MEME program was used to identify the conserved motifs of strawberry MAPKs to explore structural diversity, and each motif was subsequently annotated using the InterProScan program. As shown in Fig 4, a total of 13 conserved motifs were identified in FvMAPKs. Seven motifs (motif 1–7) were conserved in all of the FvMAPK members, among which motifs 1–5 were annotated as the protein kinase domain, indicating that all of the strawberry MAPKs were typical of the MAPK family. Motif 8 was present in all MAPK family members except FvMAPK6. All the members identified in the same group shared similar conserved motifs. For instance, in addition to the conserved motifs, most MAPK proteins in groups A, B and D contained specific motif 9, whereas most MAPKs in group D contained specific motif 10 at the N-terminal region, motifs 11 and 12 at the C-terminal region and motif 13 in the middle of the kinase domain. Interestingly, motif 7 in groups A, B and C was located in the N-terminal region, while motif 7 in group D was located in the C-terminal region.

Gene structure divergence plays a crucial role in the evolution of gene families and provides valuable evidence to evaluate phylogenetic relationships. To determine the exon-intron organization of *FvMAPK* genes, we aligned the CDSs with their corresponding genomic sequences. We found that the exon-intron organization and intron phases in the strawberry *MAPK* gene families were conserved within the same group but were strikingly divergent between different groups (Fig 4). The *FvMAPK* members in group A contained 5–6 exons and 4–5 introns; *FvMAPKs* in group B contained 6 exons and 5 introns, while those of group C had only 2 exons and 1 intron with a strictly conserved size. Group D had a higher number of exons with variable exon lengths compared with the other groups, and the number of exons ranged from 10 to 11. Exon-intron organizations in group D were the most complex and diverse, which was in accordance with the motifs of this group. Additionally, most members within the same group shared similar exon-intron organization, which further supports the classification of the *FvMAPK* genes in this study (Fig 4).

### Analysis of stress-related *cis*-regulatory elements in the *FvMAPK* promoters

**Fig 5:**
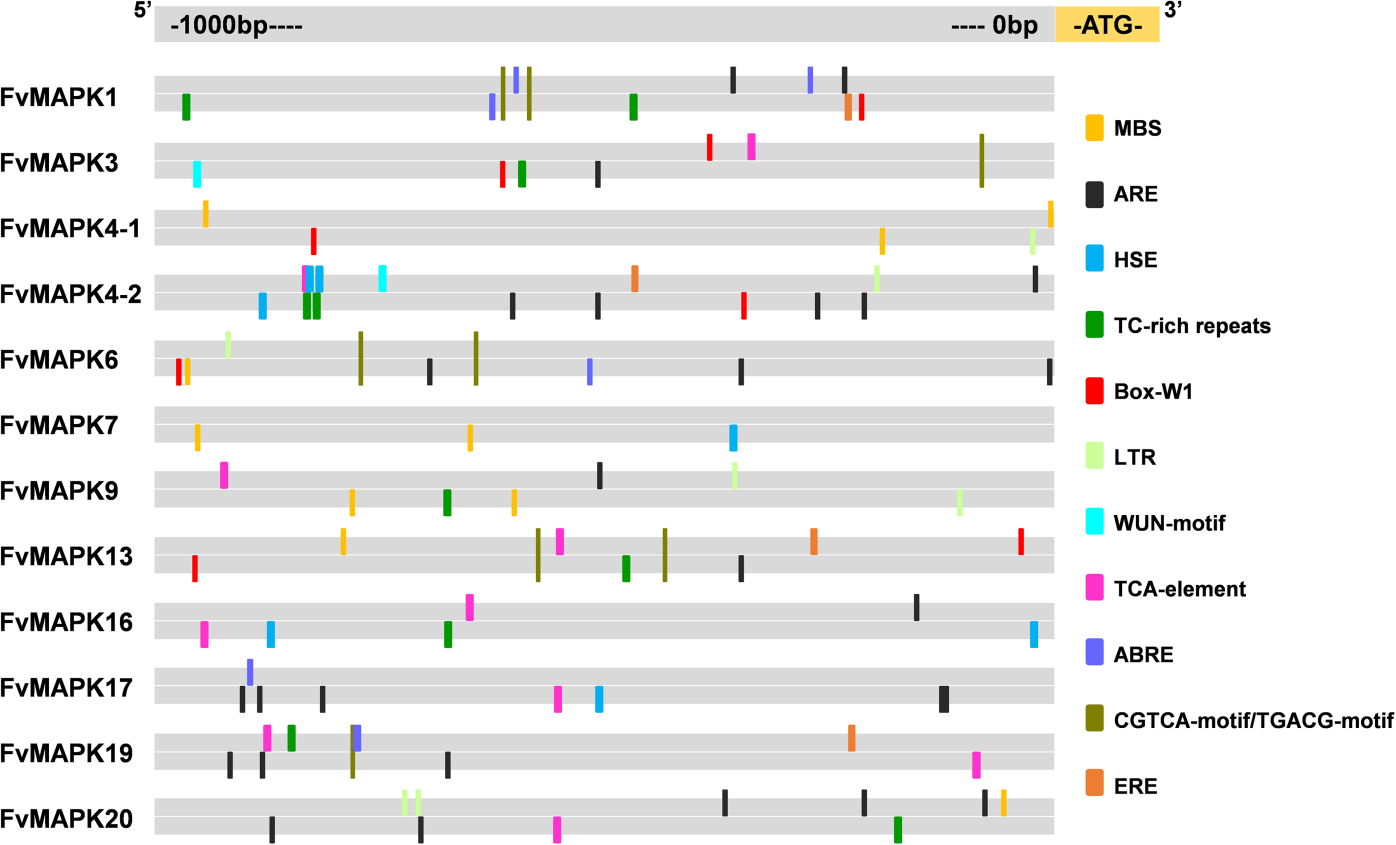
*Cis*-regulatory element analysis of the promoter regions of strawberry *MAPK* genes. Different *cis*-regulatory elements are indicated by different color round-corner rectangles and placed in their relative position on the promoter. Round-corner rectangles presented above the line indicate the forward strand of DNA, while those below indicate the reverse strand (detailed results shown in S2 Table).

Identification and analysis of *cis*-regulatory elements in promoter sequences are important features for understanding gene function and transcriptional regulation. We investigated the promoter regions (1-kb genomic DNA sequence upstream of the translation start site) of the *FvMAPK* gene family for several well-studied stresses/stimuli-related response *cis*-regulatory elements. Eleven *cis*-regulatory elements were used in this study: abiotic and biotic stress response elements (MBS, ARE, HSE, LTR, WUN-motif, TC-rich repeats, and Box-W1) and hormone-responsive elements (TCA-element, ABRE, CGTCA-motif/TGACG-motif, ERE) (Fig 5, S2 Table). ARE elements essential for anaerobic induction were found in the promoter regions of all 12 *FvMAPK* genes except *FvMAPK4-1* and *FvMAPK7*. MBS involved in drought inducibility was found in 6 *FvMAPK* genes. Heat stress responsiveness (HSE) and low-temperature responsiveness (LTR) were observed in 4 and 5 *FvMAPK* genes, respectively. TC-rich repeats, a *cis*-acting element involved in defense and stress responsiveness, were observed in 8 *FvMAPK* genes, and Box-W1, a fungal elicitor responsive element, was identified in 6 *FvMAPK* genes. Additionally, the WUN-motif, a wound-responsive element, was found in *FvMAPK3* and *FvMAPK4-2*. Interestingly, the majority of *FvMAPKs* contained one or more *cis*-regulatory elements involved in the signaling pathways of salicylic acid responsiveness (TCA-element), abscisic acid responsiveness (ABRE), MeJA responsiveness (CGTCA-motif/TGACG-motif), and the ethylene responsive element (ERE) (Fig 5).

### Predicted subcellular localization of FvMAPKs

**Fig 6:**
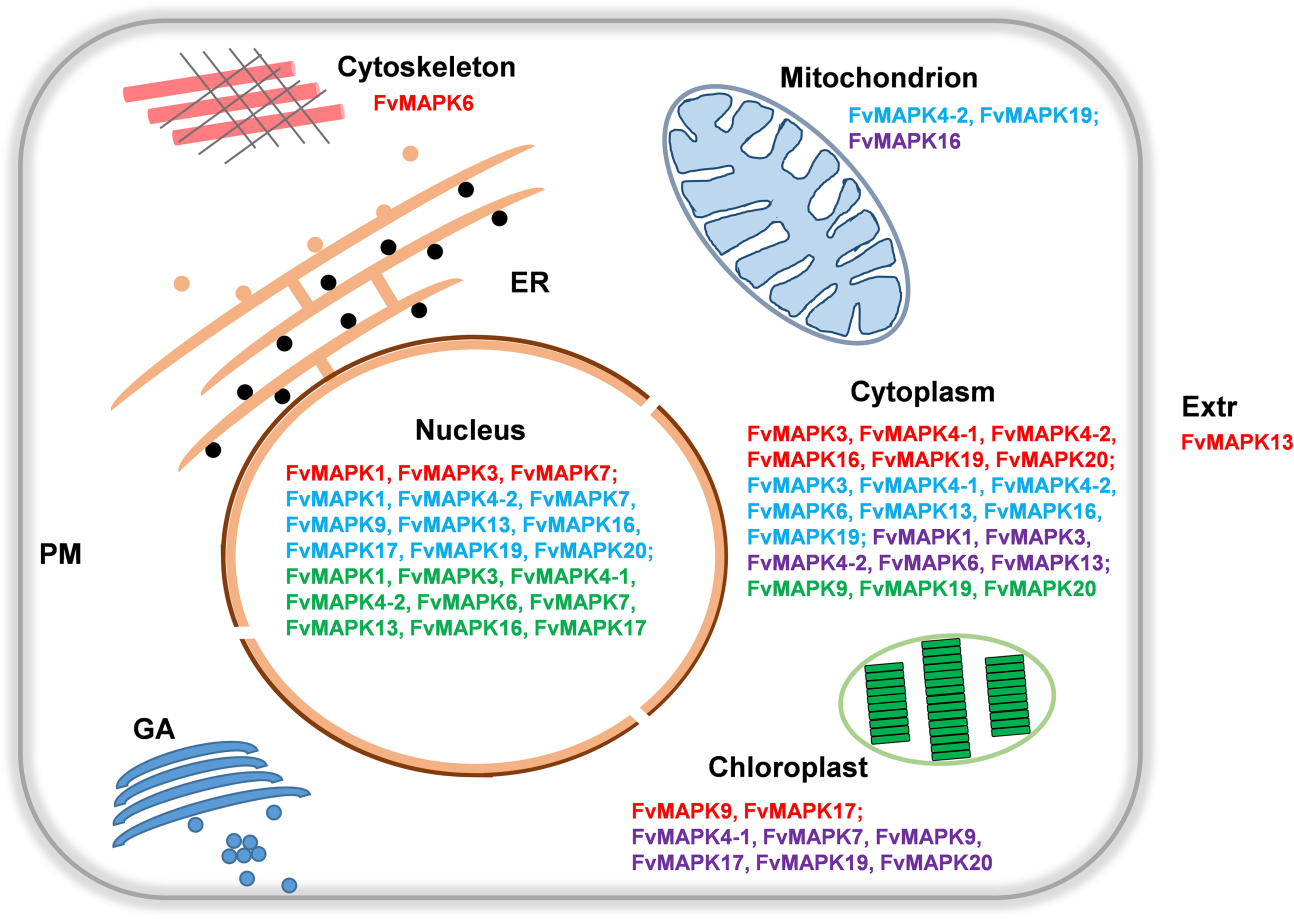
Predicted subcellular localization of the FvMAPKs. A schematic representation of the subcellular localization of the FvMAPK proteins based on four different online programs (detailed results shown in S3 Table). Red FvMAPKs indicate localization prediction by WoLF PSORT; blue FvMAPKs indicate localization prediction by CELLO v2.5; purple FvMAPKs indicate localization prediction by Plant-PLoc; green FvMAPKs indicate localization prediction by ProtComp 9.0. ER, endoplasmic reticulum; PM, plasma membrane; GA, Golgi apparatus; Extr, extracellular.

A protein’s function is usually related to its subcellular localization; therefore, the ability to predict subcellular localization directly from protein sequences will be useful for inferring protein functions. Several bioinformatics methods have been developed to predict the subcellular localization of proteins, suggesting that bioinformatics predictors can provide such information rapidly for a large number of proteins [41]. The subcellular localization of all of the predicted FvMAPK proteins was analyzed based on four different tools: WoLF PSORT [42], CELLO v2.5 [41], Plant-PLoc [43], and ProtComp 9.0 (S3 Table). As shown in Fig 6, different members localized in different subcellular compartments, such as the nucleus, cytoplasm, chloroplast, mitochondria, extracellular, and cytoskeleton. The Wolf PSORT, CELLO and ProtComp assessments revealed that most of the FvMAPK proteins were predicted to be located in the nucleus and the cytoplasm. Additionally, FvMAPK9 and 17 were located in the chloroplast, FvMAPK6 was located in the cytoskeleton, and FvMAPK13 was located in the extracellular based on the Wolf PSORT prediction. The CELLO analysis revealed that FvMAPK4-2 and 19 were predicted to be located in the mitochondria. The Plant-PLoc analysis revealed that six strawberry MAPK proteins were located in the chloroplast, five in the cytoplasm, and one in the mitochondria. These results suggest that the subcellular localization of FvMAPKs is diverse and complex. Therefore, it is worth confirming these results through further research.

### Transcript profiles of *FvMAPK* genes in different strawberry organs/tissues

To assess the potential functions of *FvMAPK* genes during strawberry development, we investigated the transcript patterns of all 12 *FvMAPK* genes in seven organs/tissues (roots, stems, runners, young leaves, mature leaves, flowers, and fruits) of the diploid woodland strawberry accession Heilongjiang-3 under normal growth conditions. Most of the *FvMAPK* genes showed no significant organ/tissue-related differences in their transcript levels, but some slight spatial differences were noted (S2 Fig). For example, *FvMAPK1* showed high transcript levels in stems, and *FvMAPK4-2* and *FvMAPK6* showed high transcript levels in young leaves. *FvMAPK16* and *FvMAPK19* shared a similar transcript profile and were transcribed at a relatively low level in roots. Additionally, *FvMAPK9* showed very low transcription levels in all ‘Heilongjiang-3’ organs/tissues.

### Transcript analysis of strawberry *MAPK* genes under different stress and hormone treatments

*MAPK* genes have been reported to play pivotal roles in the regulation of plant tolerance to various stress and related signaling transduction in various species. Hence, to investigate the potential roles of *FvMAPK* genes in response to various environmental stresses and related signaling, we exposed potted *F*. *vesca* accession Heilongjiang-3 to various artificial stress conditions (powdery mildew, cold, heat, drought, salt, ABA, Eth, MeJA, and SA) and performed semi-quantitative RT-PCR to evaluate the transcript profiles of the *FvMAPK* gene family members. Based on the results of semi-quantitative RT-PCR, six *FvMAPK* genes (*FvMAPK3*, *FvMAPK4-1*, *FvMAPK4-2*, *FvMAPK6*, *FvMAPK7*, and *FvMAPK19*) that simultaneously respond to multiple stresses were selected using RT-qPCR for further analysis and validation of their transcript abundance under various stresses.

#### Biotic stress

**Fig 7:**
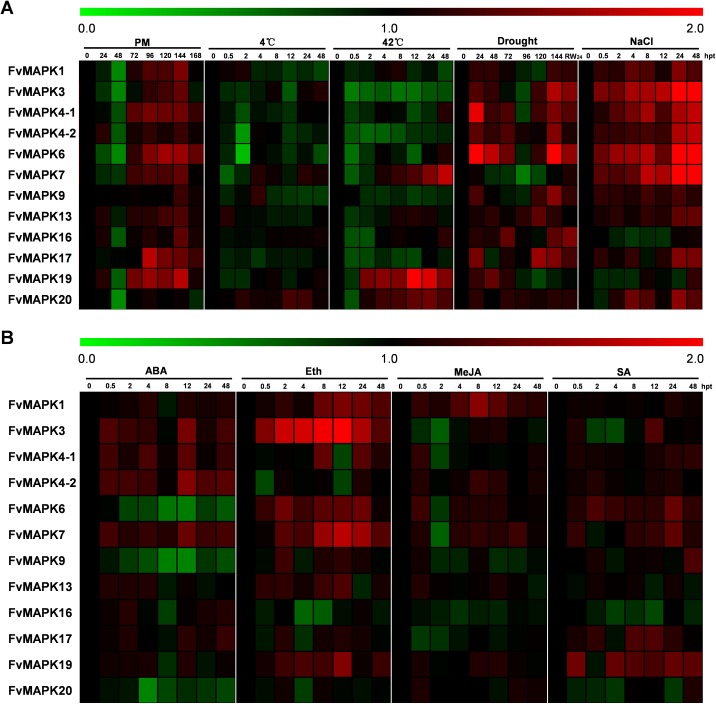
Transcript profiles of *MAPK* genes in the diploid woodland strawberry (*F*. *vesca*) during different stress treatments. Biotic and abiotic stress treatments (powdery mildew, 4°C, 42°C, drought, and salt) (A) and hormone treatments (ABA, ETH, MeJA, and SA) were applied to the plants, and the transcript profiles were generated using semi-quantitative PCR (original results shown in S3–S7 Figs). The color scale represents relative transcript levels with increased (red) or decreased (green) transcript abundance. The experiments were repeated three times, and the results were consistent.

**Fig 8:**
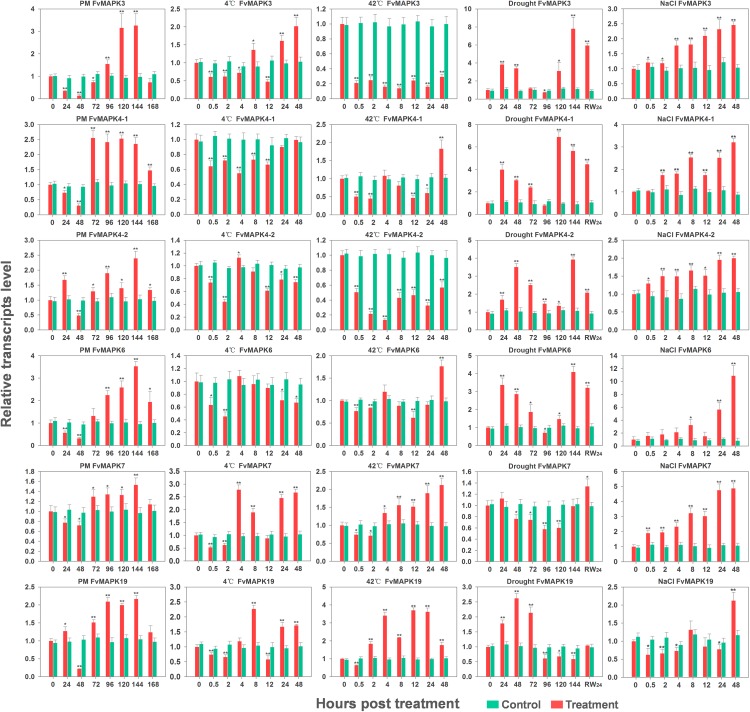
RT-qPCR analysis of six *MAPK* genes in the diploid woodland strawberry (*F*. *vesca*) in response to powdery mildew infection, 4°C, 42°C, drought, and NaCl treatments. The experiments were repeated three times, and the results were consistent. The mean values and SDs were obtained from three biological and three technical replicates. The asterisks indicate that the corresponding gene was significantly up- or down-regulated in response to treatment, as determined by Student’s t-test (* P < 0.05, ** P < 0.01).

Increasing evidence suggests that *MAPK* genes play important roles in response to pathogen infection. The biotic stress responses of the 12 strawberry *MAPK* genes were therefore investigated using semi-quantitative RT-PCR to obtain their transcript abundance under powdery mildew infection. As shown in Fig 7A, the transcript levels of most of the strawberry *MAPK* genes showed an early downregulation (24 or 48 hpi) but subsequent upregulation (72–168 hpi) in response to powdery mildew infection, while only *FvMAPK20* transcript levels showed downregulation at 48 hpi and then remained at normal levels. For example, the transcript levels of *FvMAPK4-2* and *FvMAPK19* were upregulated at 24 hpi and subsequently downregulated at 48 hpi, followed by rapid upregulation, reaching a peak at 144 hpi (2.4-fold and 2.2-fold, respectively). By contrast, *FvMAPK3*, *FvMAPK4-1*, *FvMAPK6*, and *FvMAPK7* were significantly downregulated in the earlier stage (24–72 hpi), after which the transcript levels increased rapidly and remained at a high level during the 72–144 hpi time period (Fig 8). These results suggest the potential involvement of *FvMAPK* genes in response to powdery mildew inoculation, which supports their playing an important role in biotic stress.

#### Abiotic stress

Previous work showed that *MAPKs* participate in abiotic stress responses, including responses to salt, osmotic and temperature stress. In this study, we used NaCl treatment, drought treatment, and incubation at low or high temperature (4°C or 42°C) to understand how *FvMAPKs* respond to these abiotic stresses at the transcriptional level. As shown in Fig 7A, almost all of the *FvMAPK* genes were downregulated to a greater extent by the extreme temperature treatments, while almost all of the *FvMAPK* genes were upregulated in response to the drought and NaCl treatments.

In the 4°C treatment, only some *FvMAPK* gene transcript levels (*FvMAPK7* and *FvMAPK20*) were up-regulated, while ten genes showed down-regulation (Fig 7A). For example, the transcript levels of *FvMAPK3* and *FvMAPK19* showed significant downregulation during the earlier stage (0.5–2 or 4 hpt) but were subsequently rapidly upregulated. The transcript levels of *FvMAPK4-1*, *FvMAPK4-2* and *FvMAPK6* were significantly downregulated at 2 and 4 hpt. By contrast, the transcript levels of *FvMAPK7* reached a peak of 7.7-fold at 4 hpt and then remained at a high level (Fig 8). In the 42°C treatment (Figs 7A and 8), we found that nine genes were downregulated. Among the downregulated genes, the transcript level of *FvMAPK3* was prominent and rapidly decreased to a significantly low level at 0.5 hpt, remaining at a much lower mRNA level throughout the entire treatment period, similar to the expression pattern of *FvMAPK4-2*. *FvMAPK4-1* and *FvMAPK6* were primarily down-regulated in response to the 42°C treatment and were then up-regulated (1.8-fold) at 48 hpt. On the contrary, three genes were up-regulated. The transcript level of *FvMAPK7* was slightly downregulated at the earlier stage (0.5–2 hpt) and then showed a gradual increase to 2.1-fold from 4 to 48 hpt. The transcript level of *FvMAPK19* was strongly up-regulated 2–48 h after exposure to the 42°C treatment.

After the drought treatment, 11 of the 12 *MAPK* genes were upregulated, and only one gene was downregulated (Fig 7A). Interestingly, the majority of the responsive *MAPK* genes showed a similar pattern, i.e., their transcript abundance indicated an early upregulation and subsequent downregulation, followed by a rapid upregulation. For example, *FvMAPK3* and *FvMAPK4-1* reached a peak of nearly 4-fold at 24 h post treatment (hpt) and subsequently decreased to nearly normal transcript levels at 96 hpt, after which their transcript levels rapidly increased to ~7-fold at 144 or 120 hpt, similar to *FvMAPK4-2* and *FvMAPK6*. Additionally, *FvMAPK19* was up-regulated in the earlier stage of treatment (24–72 hpt) but returned to the baseline or was down-regulated after 72 hpt. In particular, the transcript levels of *FvMAPK7* decreased continuously to significantly low levels at 96 and 120 hpt but increased to 1.4-fold when the plants were rewatered (Fig 8). Following the salt treatment, the transcript levels of ten genes were upregulated, while two genes showed the opposite pattern (Fig 7A). Of the ten up-regulated genes, most of the *FvMAPK* genes exhibited a steady or gradual increase throughout the entire salt treatment period. For example, *FvMAPK4-1*, *FvMAPK6* and *FvMAPK7* exhibited upregulated transcript levels after the salt treatment, reaching the highest levels (~3–11-fold) at 48 hpt, similar to the transcript patterns of *FvMAPK3* and *FvMAPK4-2*. However, the transcript level of *FvMAPK19* was primarily down-regulated in response to the salt treatment but rapidly increased (to 2.1-fold) at the late stage of treatment (Fig 8).

#### Hormone treatment

Phytohormones such as abscisic acid (ABA), salicylic acid (SA), methyl jasmonate (MeJA), and ethylene (Eth) play well-established roles in plant biotic and abiotic stress signaling networks. ABA is known to play an important role in plant response to various abiotic stresses [44], and analysis of transcript levels from ‘Heilongjiang-3’ leaves treated with exogenous ABA indicated that nine *FvMAPK* genes were up-regulated at different times following treatment, whereas three of the analyzed genes exhibited decreased transcript levels (Fig 7B). We also evaluated the effects of SA, MeJA, and Eth on the *FvMAPK* gene transcript profiles since SA, MeJA and ETH are involved in plant response to biotic stresses. Analysis of transcript levels from ‘Heilongjiang-3’ strawberry leaves sprayed with SA showed that six *FvMAPK* genes were up-regulated to different degrees upon treatment, whereas 2 *FvMAPK* genes were down-regulated (Fig 7B). The transcript level of *FvMAPK3* showed an early upregulation and subsequent downregulation, but final upregulation, while the *FvMAPK1*, *FvMAPK9* and *FvMAPK13* transcript levels remained unchanged (Fig 7B). Following treatment with MeJA, five genes were upregulated and four genes were downregulated. The three other genes showed an early upregulation but subsequent downregulation (Fig 7B). Similar to the Eth treatment, most *FvMAPK* genes showed altered transcription in leaves following treatment. Of these, seven *FvMAPK* genes exhibited increased transcript levels, while three genes were down-regulated. *FvMAPK9* and *FvMAPK17* transcript abundance showed an initial decrease but a subsequent increase (Fig 7B).

**Fig 9:**
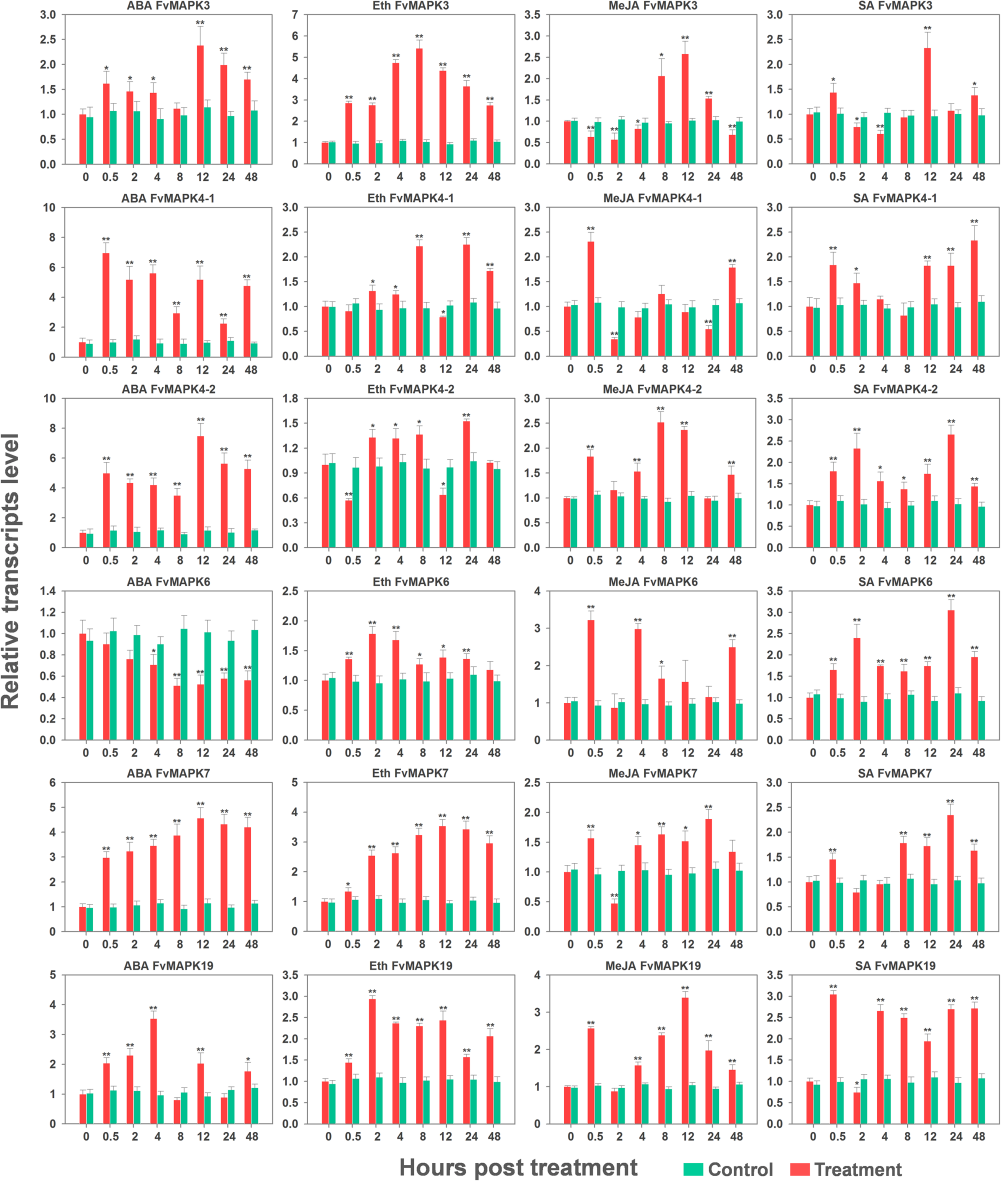
RT-qPCR analysis of six *MAPK* genes in the diploid woodland strawberry (*F*. *vesca*) in response to ABA, Eth, MeJA, and SA treatments. The experiments were repeated three times and yielded consistent results. The mean values and SDs were obtained from three biological and three technical replicates. The asterisks indicate that the corresponding gene was significantly up- or down-regulated in response to treatment, as determined by Student’s t-test (* P < 0.05, ** P < 0.01).

RT-qPCR analysis revealed that the transcript level of *FvMAPK3* was highly induced during the early stage (0.5 hpt to 4 hpt) and peaked during the middle stage (8–12 hpt) in response to the ABA and Eth treatments; however, the transcript level was significantly down-regulated during the early stage and then increased to more than 2-fold at 12 hpt in response to both the MeJA and SA treatments (Fig 9). The transcript level of *FvMAPK4-1* was significantly upregulated during the early stage (0.5 hpt) in response to ABA, MeJA and SA and was significantly induced during the late stage following treatment with Eth (Fig 9). *FvMAPK4-2* displayed a strong response to the ABA, MeJA and SA treatments and reached a peak of 7.0-fold after ABA treatment; by contrast, an initial down-regulation within 0.5 hpt followed by fluctuation around the control value was observed in response to the Eth treatment (Fig 9). The transcript level of *FvMAPK6* was up-regulated in response to the Eth, MeJA and SA treatments but was significantly down-regulated in response to the ABA treatment. The transcript pattern of *FvMAPK7* was positively regulated following all hormone treatments, but its up-regulation was more significant following treatment with ABA (Fig 9). The transcript profile of *FvMAPK19* was similar to that of *FvMAPK7*, whereas the transcript profile of *FvMAPK19* was up-regulated in the earlier stage of the ABA stress treatment (Fig 9).

### Interaction network analysis

**Fig 10:**
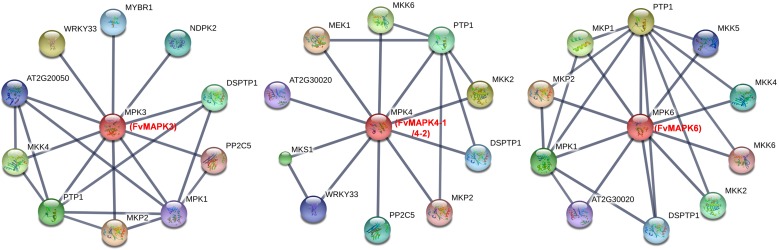
Interaction network analysis of MAPK proteins identified in strawberry and related genes in *Arabidopsis*. The line thickness relates to the combined score. The homologous genes of strawberry are presented in red font in parentheses.

Increasing evidence has confirmed that MAPK cascades are involved in a wide range of stress responses as a result of the interaction between MAPK and various other proteins, among which MPK3, MPK4, and MPK6 in *Arabidopsis* are the most extensively studied [8, 45]. Recently, interaction networks of gene families have become a very useful method to study their function [46]. To identify potential interaction networks of strawberry MAPKs, we constructed interaction networks of FvMAPK3, FvMAPK4-1, FvMAPK4-2, and FvMAPK6 based on experimentally validated interactions in *Arabidopsis* using STRING 10. There were 10 high confidence interactive proteins involved in the MPK3, MPK4, and MPK6 networks, including MAPK cascades proteins (MEK1, MKPs, and MKKs) that are involved in biotic or abiotic stress and hormone signaling, WRKY33 and MKS1 involved in the regulation of plant defense response, and PP2Cs involved in stress and defense signaling, among other functions (Fig 10, S4 Table).

## Discussion

### Identification and bioinformatics analysis of *FvMAPK* genes revealed their functional conservation and divergence

MAPKs, a family of serine/threonine protein kinases, play essential roles in mediating biotic and abiotic stresses and related signal transduction pathways [11]. To date, the features and functions of the *MAPK* gene family have been identified and investigated in several plant species, including *Arabidopsis* [6], rice [26], tomato [27], apple [29], and maize [28]. In the present study, a total of 12 *MAPKs* from *F*. *vesca* were identified. Consistent with previous results from other plant species, the 12 *MAPKs* in *F*. *vesca* possessed 11 domains (I-XI; Fig 1), and TEY or TDY motifs of *MAPKs* are located in the activation loop between kinase domain VII and VIII [6, 47]. Interestingly, more *MAPK* members from *Arabidopsis*, apple, tomato, grape and strawberry contain the TEY motif than the TDY motif. By contrast, rice and maize contain more *MAPKs* with the TDY motif than with the TEY motif (Table 2). We speculate that *MAPKs* with the TEY motif may play more critical roles than *MAPKs* with the TDY motif in eudicots plants. Moreover, MAPKs may have a CD domain, which is defined as (LH)DXXDE(P)XC (where X represents any amino acid), and the two adjacent acidic residues D (aspartate) and E (glutamate) have been shown to play critical roles in interacting with a cluster of the amino acids K (lysine) and R (arginine) in MAPKKs [48]. In our study, the members of groups A and B were found to possess a CD domain or modified CD domain in their C-terminal region, whereas groups C and D did not possess such a CD domain (Fig 2). The group distribution of the CD domain for FvMAPK proteins is in accordance with previous findings from other species, such as *B*. *distachyon* and cucumber [49, 50].

**Table 2:** The number of *MAPK* genes in *Arabidopsis*, strawberry, apple, grape, tomato, maize, and rice.

Species	Gene	TEY			TDY	No group	Total
		Group A	Group B	Group C	Group D		
*Arabidopsis*	*AtMAPK*	3	5	4	8	-	20
Strawberry	*FvMAPK*	2	3	2	5	-	12
Apple	*MdMAPK*	5	6	5	10	-	26
Grape	*VvMAPK*	2	3	2	5	2	14
Tomato	*SlMAPK*	3	4	2	7	-	16
Maize	*ZmMAPK*	4	3	2	10	-	19
Rice	*OsMAPK*	2	2	2	11	-	17

Phylogenetic tree analysis suggested that a large number of *FvMAPK* members belong to group D (Fig 1), consistent with the results from *Arabidopsis*, rice and apple [6, 26, 29]. Our results support the previous conclusion that group D expanded before and after the divergence of monocots and dicots [5]. Additionally, strawberry *MAPK* genes are more closely related to genes from apple *MAPK* genes, suggesting that strawberry and apple have a close evolutionary relationship, which is consistent with the fact that strawberry and apple are both members of *Rosaceae* [51]. A comparison of exon-intron organization indicated that the A, B and C groups share a similar number of exons, and the lengths of exons are more conserved. However, members in the D group have more exons, and the lengths of these exons are diverse (Fig 4). The consistency of the domain organization and conserved motifs of the FvMAPK proteins with phylogenetic subclasses further supported the close evolutionary relationships among FvMAPKs, which might be essential for functional conservation. Additionally, the complexity of the FvMAPK proteins subcellular localization might reveal their functional diversity and variety (Fig 6).

### Strawberry *MAPK* genes are putatively involved in a range of biological processes

It has been reported that plant MAPK genes play essential roles in the regulation of developmental processes [25]. A study on the apple MAPK gene family showed that all of the selected genes were expressed in at least one of the tissues tested [29]. Our semi-quantitative RT-PCR results showed that most of the *FvMAPK* genes showed no significant transcript difference among organs/tissues (S2 Fig), indicating these *MAPKs* are likely to play a ubiquitous role in strawberry development. It is worth noting that *FvMAPK4-2* was transcribed at a high level in young leaves (S2 Fig), indicating its potential role in the development of young strawberry leaves.

Accumulating evidence has demonstrated that the *MAPK* genes are involved in regulating the pathogen-induced defense system and responses to biotic and abiotic stresses and also play a central role in related signal transduction pathways [8, 10]. In our study, a high number of stress-related (e.g., drought, extreme temperatures, high salinity, wounding, and disease) and hormone-related (e.g., abscisic acid, ethylene, and salicylic acid) *cis*-regulatory elements were found in the putative promoter regions of the *FvMAPK* gene (Fig 5), indicating that *FvMAPK* genes might have potential functions in stress adaptations and various hormone signaling pathways. Additionally, our semi-quantitative RT-PCR and RT-qPCR data revealed an abundance of *FvMAPK* gene transcripts in response to multiple treatments and a special transcript profile of tissues (Figs 7–9).

Recently, many studies have focused on *MAPK* genes in groups A and B, and the genes in these two groups have been widely studied. *AtMPK3* and *AtMPK6* of group A as well as their orthologs in other plant species have been strongly associated with numerous biotic and abiotic stresses [13]. In *Arabidopsis*, flagellin *flg22* may trigger a complete MAPK cascade MEKK1-MKK4/5-MAPK3/6, which in turn increases the expression of *WRKY22/29* and confers resistance to both bacterial and fungal pathogens [14]. Additionally, *AtMPK3* and *AtMPK6* are induced transcriptionally by various environmental stresses and hormone responses [10, 45]. *OsMPK5*, a group A MAPK in rice (*Oryza sativa*), is also induced by ABA as well as various biotic (fungal and bacterial infections) and abiotic (wounding, drought, salt and cold) stresses. Gain and loss-of-function analyses revealed that *OsMPK5* positively regulates abiotic stress tolerance and negatively regulates *PR* gene expression and broad-spectrum disease resistance in rice. In our study, *FvMAPK3* and *FvMAPK6* were significantly induced by powdery mildew infection during the late stage (120–144 hpi) and accumulated to high levels in response to drought, salt, SA, Eth and MeJA treatments (Figs 8 and 9), consistent with the transcript profiles of *AtMPK3*, *AtMPK6* and *OsMPK5*. Interaction networks showed that FvMAPK3 and FvMAPK6 had stronger interactions with MKK4 and MKK5 (Fig 10). Thus, we speculate that the dramatically increased expressions of *FvMAPK3* and *FvMAPK6* in response to the range of treatments may have important implications for stress signaling and may also mediate the crosstalk between different signaling pathways, especially in pathogen resistance. Xing [20] demonstrated that AtMKK1–AtMPK6 was a key module in an ABA-dependent signaling cascade that caused H_2_O_2_ production and stress responses. However, the *FvMAPK6* transcript abundance decreased after exogenous ABA treatment (Fig 9), which implies different roles of *FvMAPK6* and *AtMPK6* during ABA signaling in strawberry.

Group B MAPKs, represented by *Arabidopsis MPK4* and *MPK11*, are implicated in pathogen defense and abiotic stress responses [13]. A complex composed of MPK4, its nuclear substrate MKS1, which acts downstream of MPK4 in the SA-dependent pathway and interacts with the transcription factor WRKY33. Challenge with *Pseudomonas syringae* or flagellin treatments activates MPK4, which phosphorylates MKS1 and releases WRKY33. The released WRKY33 targets the promoter of *PAD3* that encodes an enzyme required for the synthesis of camalexin, which indicates that the MEKK1–MKK1/MKK2–MPK4–MKS1/WRKY33 cascade negatively regulates innate immune responses in plants [15–17, 52]. These findings are consistent with the results observed for *AtMPK4*, *FvMAPK4-1* and *FvMAPK4-2*, which displayed high transcript levels after infection with strawberry powdery mildew, accumulated to high levels during SA treatment (Figs 8 and 9), and had stronger interactions with the MKK2, MKS1 and WRKY33 proteins (Fig 10). Therefore, it is reasonable to speculate that *FvMAPK4-1* and *FvMAPK4-2* mediate crosstalk between different signaling pathways and play crucial roles in the acquired immune response to the biotrophic fungus *Podosphaera aphanis*. MPK4 and MPK6 in *Arabidopsis* are phosphorylated and activated by MKK2, which is specifically activated by cold and salt stress [18]. Similarly, *FvMAPK4-1*, *FvMAPK4-2* and *FvMAPK6* were highly induced by salt stress in the current study (Fig 8), indicating that these genes might participate in similar pathways as *AtMPK4* and *AtMPK6* in response to salt stress. Intriguingly, *FvMAPK4-1*, *FvMAPK4-2* and *FvMAPK6* were insensitive to cold stress (Fig 8). Research has demonstrated the rapid and transient activation of *AtMPK4* and *AtMPK6* by low temperature, low humidity, hyper-osmolarity, touch and wounding, and activation of *AtMPK4* and *AtMPK6* is associated with tyrosine phosphorylation but not with the amounts of mRNA or protein [53]. Thus, we speculate that activation of FvMAPK4-1, FvMAPK4-2 and FvMAPK6 protein kinase activities might not be correlated with their transcript or protein levels following cold stress.

The functions of *MAPKs* in groups C and D are less clear at present. Recently, all group C *MPKs* in *Arabidopsis*, *MPK1*, *MPK2*, *MPK7* and *MPK14*, were shown to act downstream of *AtMKK3* and play an important role in plant immune and stress responses [54]. In this study, *FvMAPK1* and *FvMAPK7* showed an increased transcript abundance following the powdery mildew and hormone treatments (Figs 7–9), which suggests that these *MAPK* genes might have similar functions in pathogen defense. The *MAPK* genes of group D have not been as well studied as those of group A, but previous studies have shown that *AtMPK9* is preferentially and highly expressed in guard cells and functions as a positive regulator downstream of ROS in guard cell ABA signaling [55]. However, the transcription level of *FvMAPK9* was downregulated by the ABA treatment (Fig 9), which suggests that *FvMAPK9* might have functions that differ from *AtMPK9* in ROS-mediated ABA signaling. *ZmMAPK17*, a novel maize group D MAP kinase gene, is involved in the response to exogenous signaling molecules, such as ABA, H_2_O_2_, SA, JA and Eth, and is induced by low temperature and osmotic stress, and *ZmMPK17*-overexpressing plants displayed enhanced resistance to viral pathogens [24]. This report is consistent with our data, which showed that the transcript level of *FvMAPK17* was induced by multiple stress responses, such as powdery mildew, drought, salt, ABA, Eth and MeJA treatments (Fig 7). These results indicate that *FvMAPK17* genes might have functions similar to *ZmMPK17* and are involved in the signaling response to various hormones. It is notable that *FvMAPK19* was highly induced by the powdery mildew, abiotic stress (heat and drought), and all hormone (ABA, Eth, MeJA, and SA) treatments (Figs 8 and 9). Therefore, we speculate that *FvMAPK19* might play an important role in the response to biotic and abiotic stresses and may also be involved in responses to diverse hormone signaling pathways and the crosstalk among these pathways.

In conclusion, the present study provides comprehensive information about the phylogenetic relationships, protein domains, exon-intron organizations, conserved motifs, *cis*-acting elements, subcellular localization, interaction networks, and transcript profile analysis of *MAPK* genes in strawberry. The responsiveness of the *FvMAPK* genes to a wide range of biotic and abiotic stresses as well as hormone treatments suggests that they are involved in the tolerance of strawberry to environmental stresses. Further studies are needed to advance the understanding of the functions of the *FvMAPK* genes in strawberry, an economically important fruit crop.

## Supporting information

S1 Fig Motif sequences of FvMAPK proteins identified using MEME tools. (TIF)

S2 Fig Transcript profiles of 12 *FvMAPK* genes in different tissues analyzed by using semi-quantitative PCR. *FvRib413* and *FvGAPDH2* were used as internal control. Lanes: R: roots, S: stems, RN: runners, YL: young leaves, ML: mature leaves, FL: flowers, F: fruits.
(TIF)

S3 Fig Transcript profiles of 12 *FvMAPK* genes under powdery mildew inoculation analyzed by using semi-quantitative RT-PCR. *FvRib413* and *FvGAPDH2* were used as internal control.
(TIF)

S4 Fig Transcript profiles of 12 *FvMAPK* genes under 4°C and 42°C treatments analyzed by using semi-quantitative RT-PCR. *FvRib413* and *FvGAPDH2* were used as internal control.
(TIF)

S5 Fig Transcript profiles of 12 *FvMAPK* genes under drought and NaCl treatments analyzed by using semi-quantitative RT-PCR. *FvRib413* and *FvGAPDH2* were used as internal control.
(TIF)

S6 Fig Transcript profiles of 12 *FvMAPK* genes under ABA and ETH treatments analyzed by using semi-quantitative RT-PCR. *FvRib413* and *FvGAPDH2* were used as internal control.
(TIF)

S7 Fig Transcript profiles of 12 *FvMAPK* genes under MeJA and SA treatments analyzed by using semi-quantitative RT-PCR. *FvRib413* and *FvGAPDH2* were used as internal control.
(TIF)

S1 Table The primers used for the *FvMAPK* genes in this study. (DOC)

S2 Table Predicted stress-related *cis*-acting elements in the promoter regions of *FvMAPKs*. (XLS)

S3 Table Predicted subcellular localization of the FvMAPKs derived from four different programs. (DOC)

S4 Table Annotation summary of proteins involved in the FvMAPKs interaction network. (XLS)
