# Supplementary figures and images for: Bioinformatics identification and transcript profile analysis of the mitogen-activated protein kinase gene family in the diploid woodland strawberry Fragaria vesca
Source: PLoS One. 2017 May 31;12(5):e0178596. doi: 10.1371/journal.pone.0178596 (PMC5451138; doi:10.1371/journal.pone.0178596)

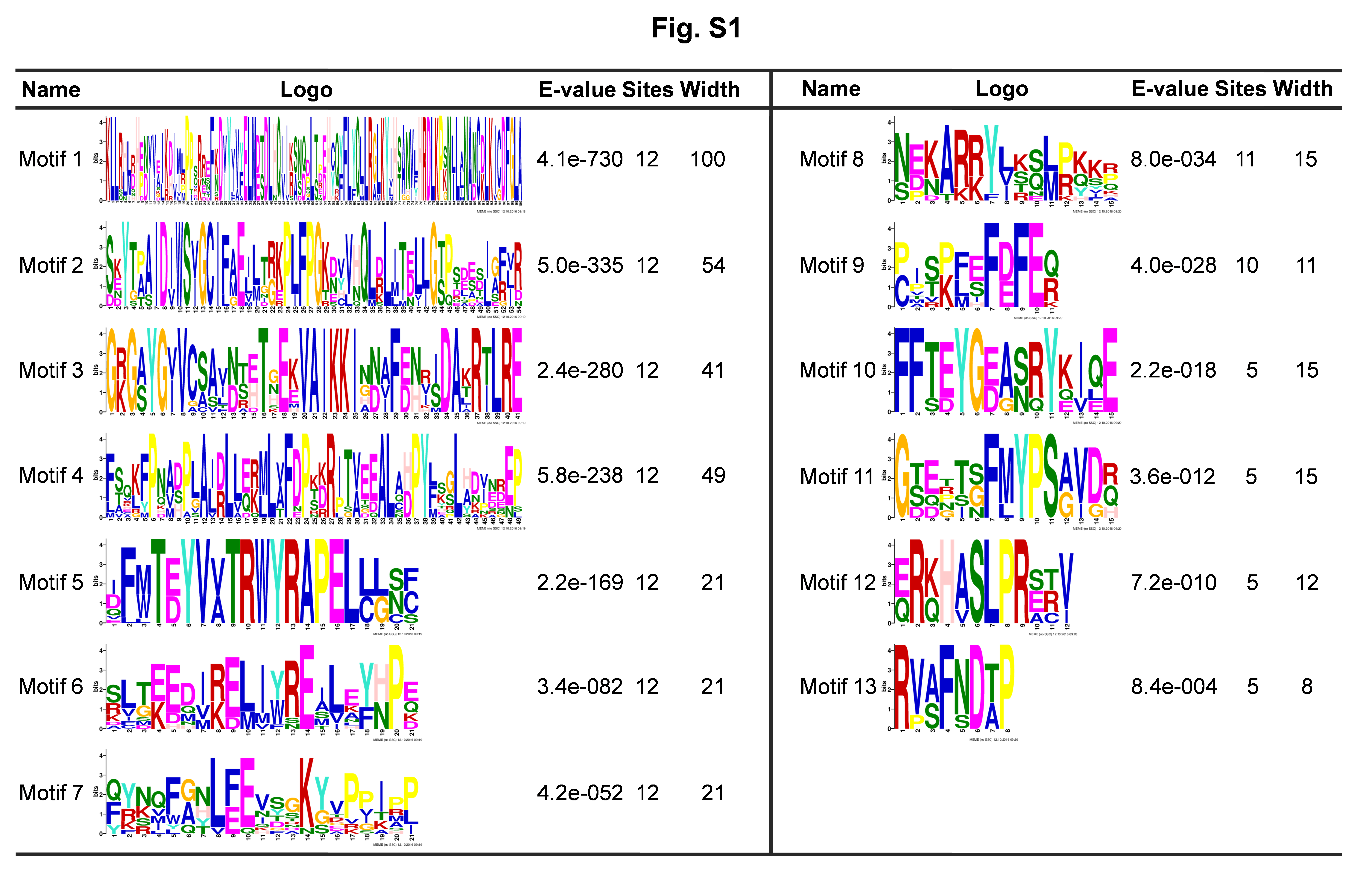

Supplement: S1 Fig — (TIF) [file pone.0178596.s001.tif]

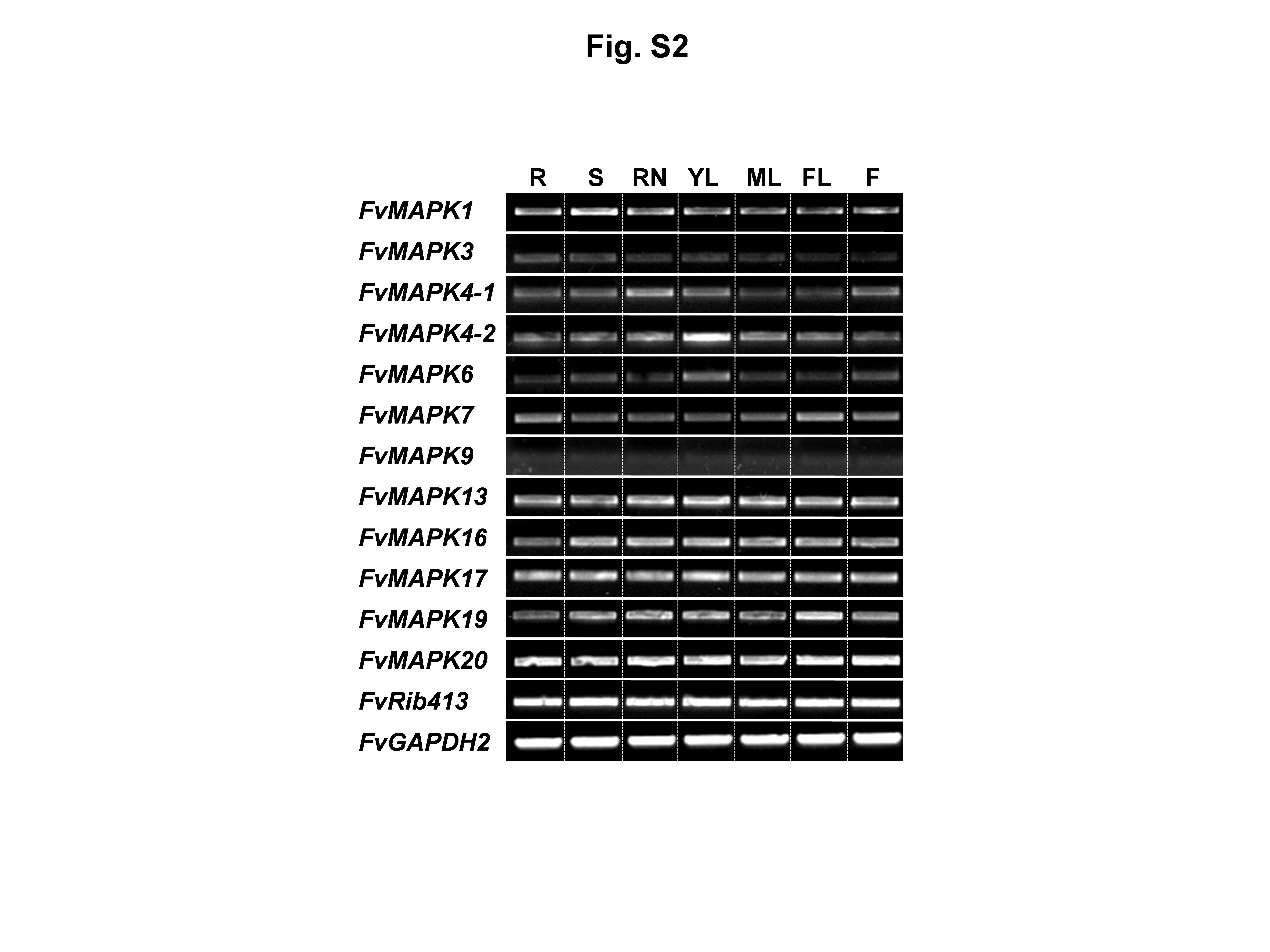

Supplement: S2 Fig — FvRib413 and FvGAPDH2 were used as internal control. Lanes: R: roots, S: stems, RN: runners, YL: young leaves, ML: mature leaves, FL: flowers, F: fruits. (TIF) [file pone.0178596.s002.tif]

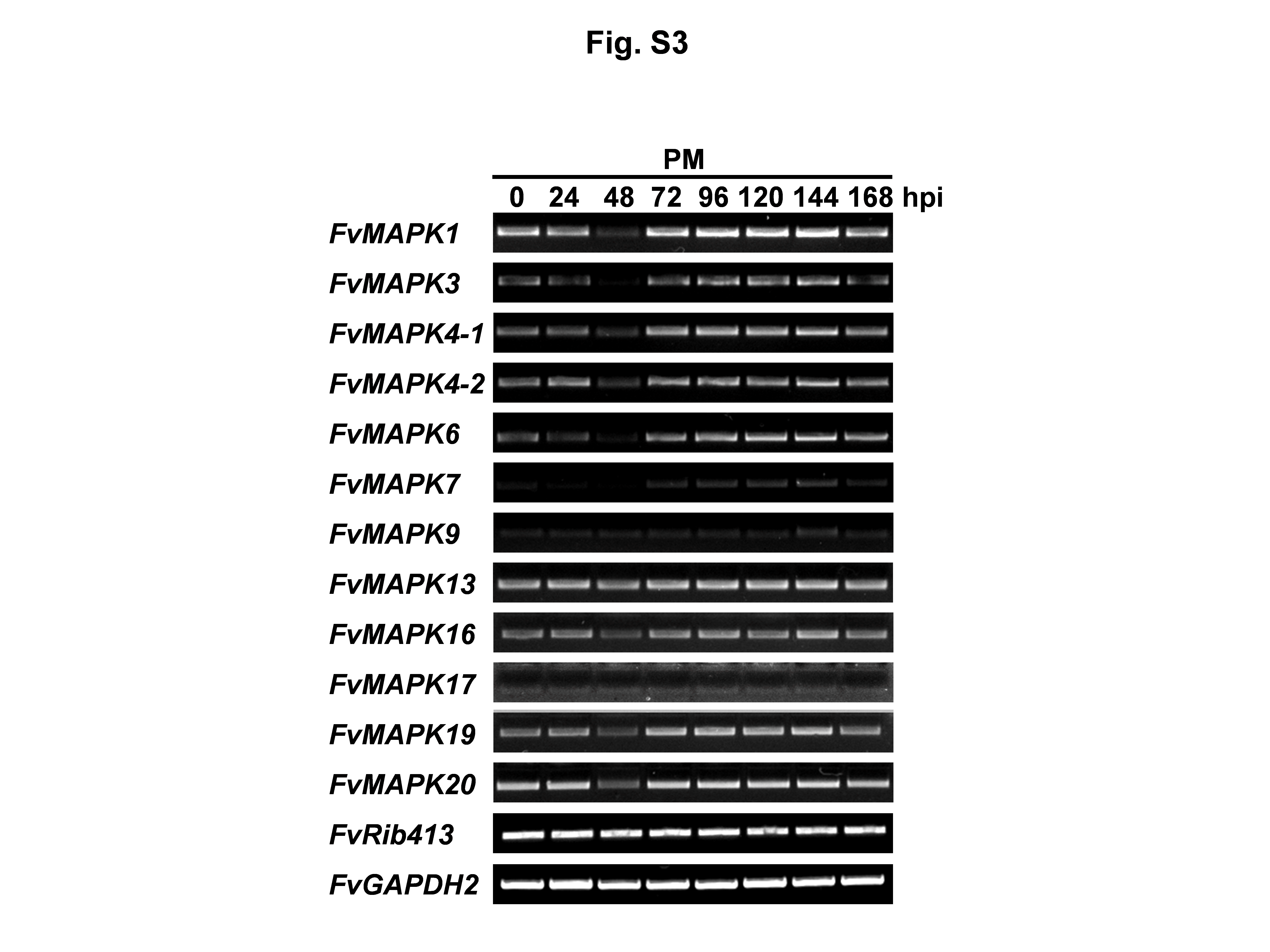

Supplement: S3 Fig — FvRib413 and FvGAPDH2 were used as internal control. (TIF) [file pone.0178596.s003.tif]

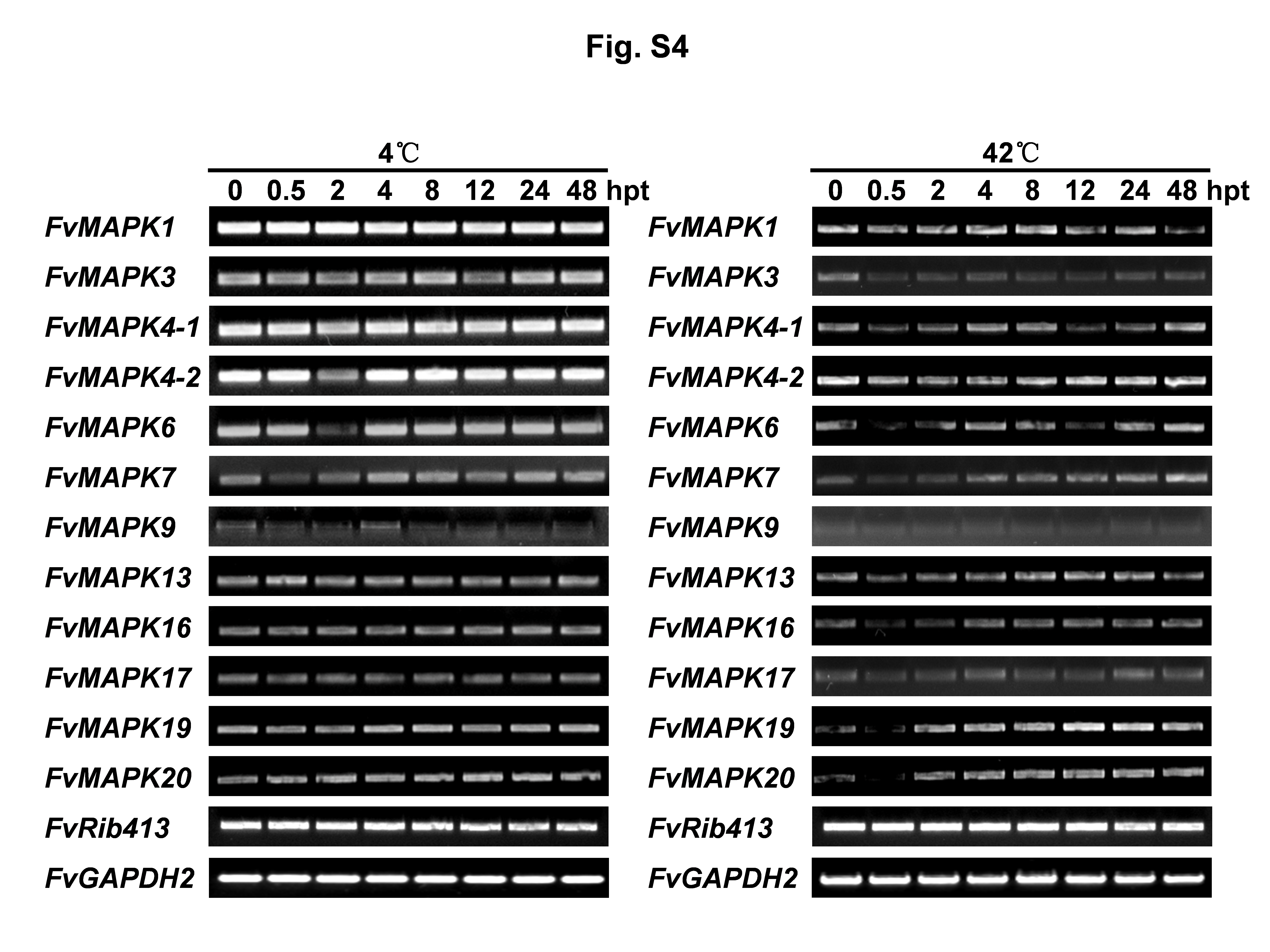

Supplement: S4 Fig — FvRib413 and FvGAPDH2 were used as internal control. (TIF) [file pone.0178596.s004.tif]

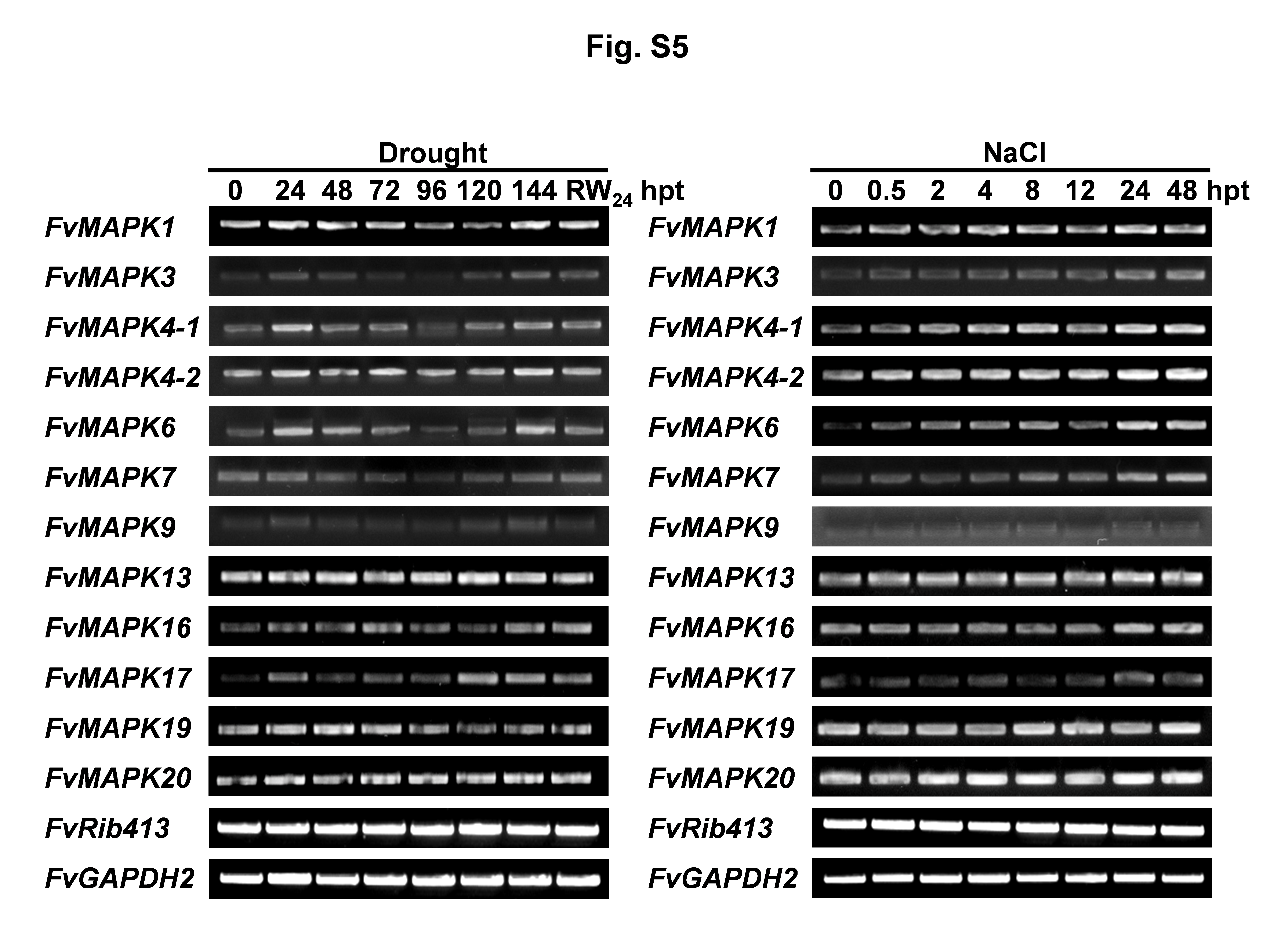

Supplement: S5 Fig — FvRib413 and FvGAPDH2 were used as internal control. (TIF) [file pone.0178596.s005.tif]

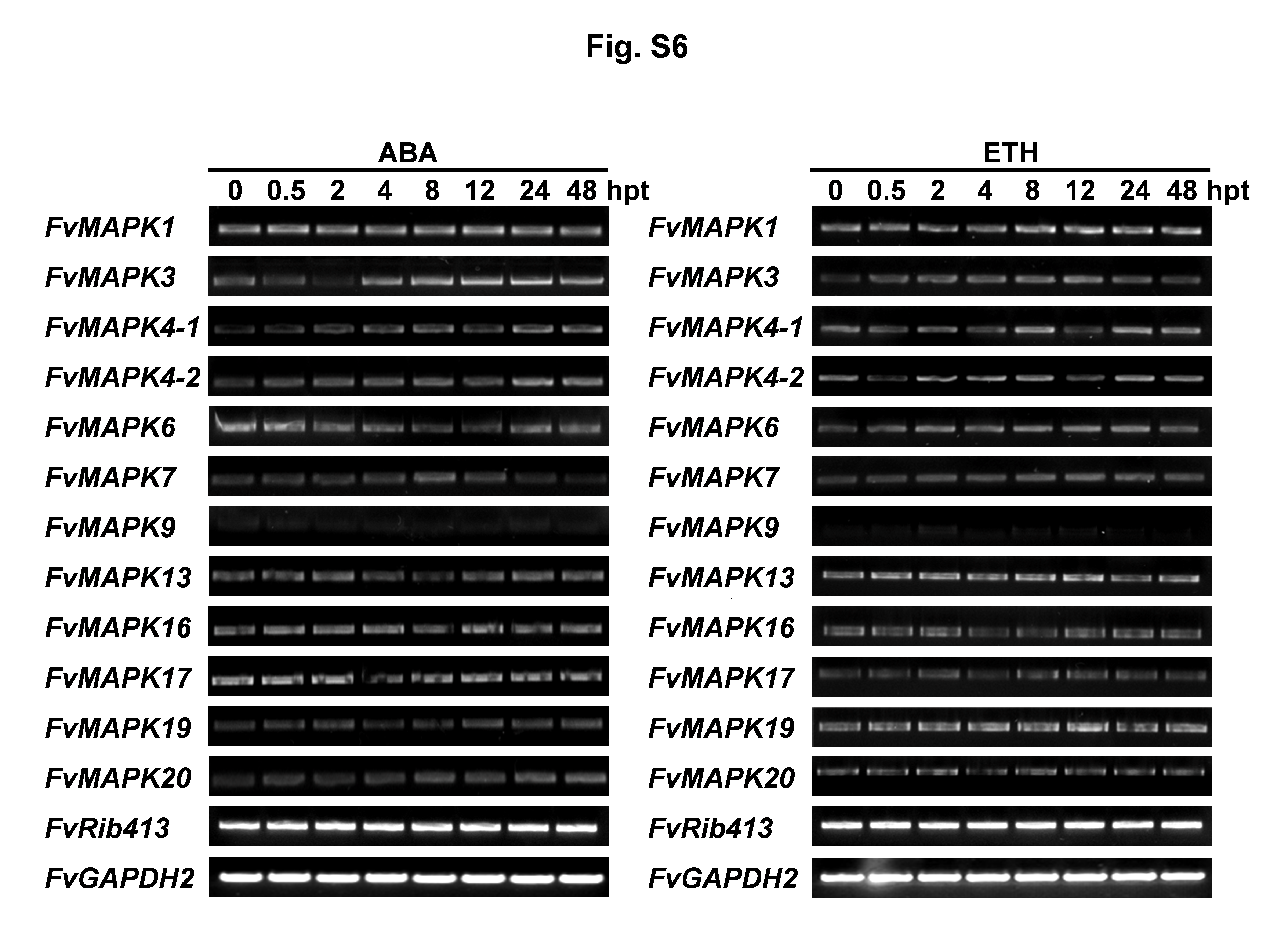

Supplement: S6 Fig — FvRib413 and FvGAPDH2 were used as internal control. (TIF) [file pone.0178596.s006.tif]

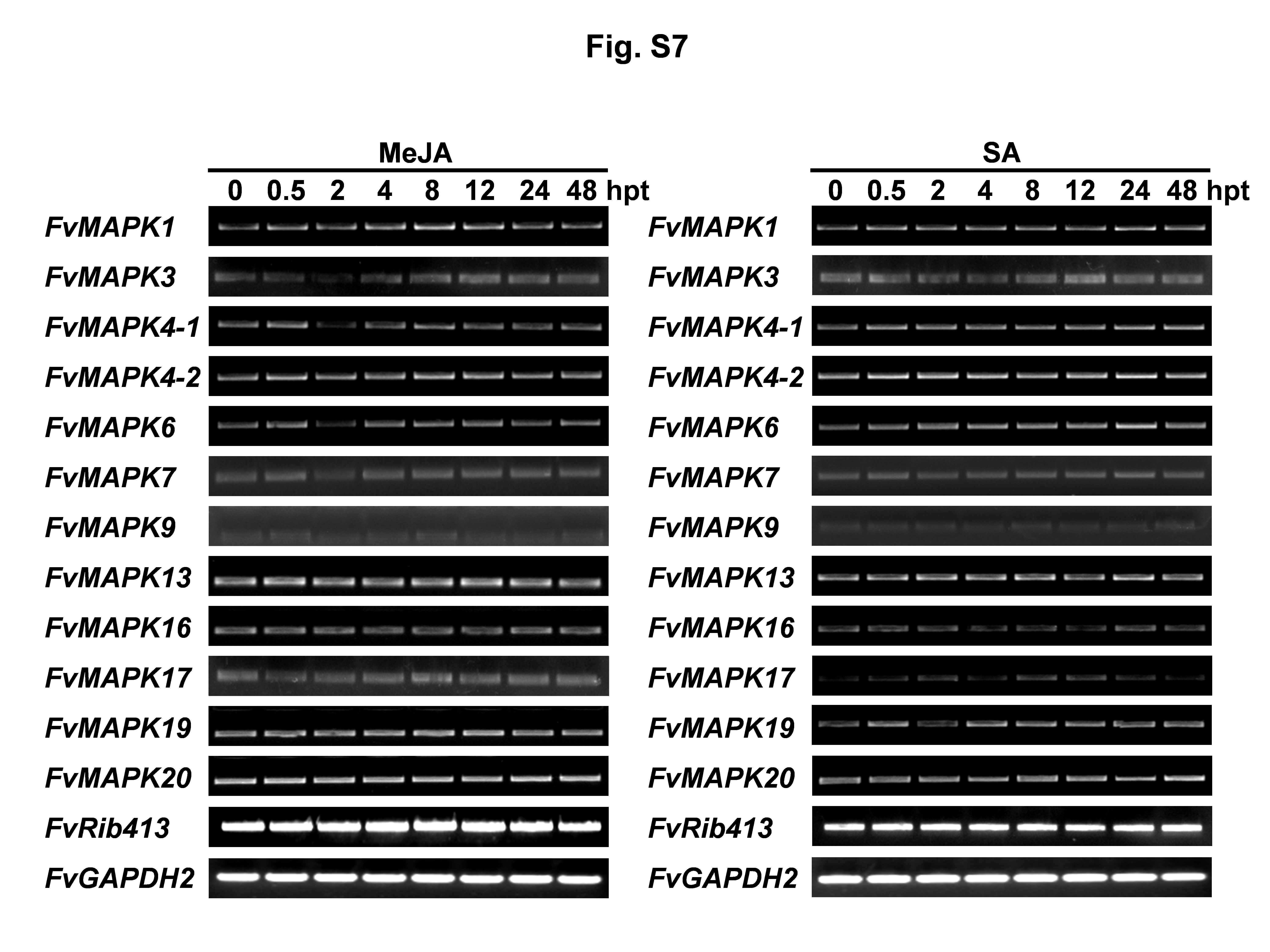

Supplement: S7 Fig — FvRib413 and FvGAPDH2 were used as internal control. (TIF) [file pone.0178596.s007.tif]
