# Supplementary material for: Bioinformatics identification and transcript profile analysis of the mitogen-activated protein kinase gene family in the diploid woodland strawberry Fragaria vesca
Source: PLoS One. 2017 May 31;12(5):e0178596. doi: 10.1371/journal.pone.0178596 (PMC5451138; doi:10.1371/journal.pone.0178596)
Supplement: S1 Table — (DOC) [file pone.0178596.s008.doc]

**Supplementary Table S1.** The primers used for the *FvMAPK* genes in this study.

| **Primer Names** | **Sequence (5'-3')** | **Description^1^** |
| --- | --- | --- |
| FvMAPK1-qF | GTATGATCCGAATGGTAACCCTCCC | RT-qPCR |
| FvMAPK1-qR | AGGAATACACTTGACCGTTGCTTGC | RT-qPCR |
| FvMAPK3-qF | TTCGAGATCACCAACAAGTACCGC | RT-qPCR |
| FvMAPK3-qR | GATCGCCACCATCTCGTTCGTC | RT-qPCR |
| FvMAPK4-1-qF | TGGAGCTGACCACAACATCAGAGG | RT-qPCR |
| FvMAPK4-1-qR | GTCTCAGCGTTCACAGCAGCACA | RT-qPCR |
| FvMAPK4-2-qF | CCGATGATGCAAGTCTTGGATTTCT | RT-qPCR |
| FvMAPK4-2-qR | GTGACAAAGTGCCTCGCCAACAG | RT-qPCR |
| FvMAPK6-qF | CACCCAGCAGCAGCAGCAGG | RT-qPCR |
| FvMAPK6-qR | AATTCAAAGCCGAGCAGACGATG | RT-qPCR |
| FvMAPK7-qF | CGAATGGAATCAGGCAACGGG | RT-qPCR |
| FvMAPK7-qR | ACACCGTATGCTCCTCTCCCTATGG | RT-qPCR |
| FvMAPK9-qF | TCCACAGCAACAAACAACAAAGCC | RT-qPCR |
| FvMAPK9-qR | CAAGGATGATTCACAGCCACACGA | RT-qPCR |
| FvMAPK13-qF | AGCAGCTCCCACATGTCCCAAA | RT-qPCR |
| FvMAPK13-qR | TGAAAGGAGATGGGCAAATGGG | RT-qPCR |
| FvMAPK16-qF | TGAGCAGCGAAGGATGGTTAGGAA | RT-qPCR |
| FvMAPK16-qR | CCTTCCACTTCATTGTCTTCCCTCTC | RT-qPCR |
| FvMAPK17-qF | GTGGTCGGAGGAGGATGAAGAGTTG | RT-qPCR |
| FvMAPK17-qR | CCATAGCTACCTTTGCCAATCACTTCT | RT-qPCR |
| FvMAPK19-qF | ACGCAGTTCTTCCTCAGCAGTCTATCTC | RT-qPCR |
| FvMAPK19-qR | CGGGTGGTATGGGTTGGTGTTG | RT-qPCR |
| FvMAPK20-qF | TTCTCCTCGCAAACCAAGCAAGC | RT-qPCR |
| FvMAPK20-qR | TGTAGCAGCAGCAGAACCAATCCC | RT-qPCR |
| FvRib413-qF | ACCGTTGATTCGCACAATTGGTCATCG | RT-qPCR |
| FvRib413-qR | TACTGCGGGTCGGCAATCGGACG | RT-qPCR |
| FvGAPDH2-qF | CCCAAGTAAGGATGCCCCCATGTTCG | RT-qPCR |
| FvGAPDH2-qR | TTGGCAAGGGGAGCAAGACAGTTGGTAG | RT-qPCR |

1 The type of experiment for which the primers were used is indicated in brackets (RT-qPCR: reverse transcription quantitative PCR).
