# Supplementary material for: Bioinformatics identification and transcript profile analysis of the mitogen-activated protein kinase gene family in the diploid woodland strawberry Fragaria vesca
Source: PLoS One. 2017 May 31;12(5):e0178596. doi: 10.1371/journal.pone.0178596 (PMC5451138; doi:10.1371/journal.pone.0178596)
Supplement: S3 Table — (DOC) [file pone.0178596.s010.doc]

**Supplementary Table** **S3.** Predicted subcellular localization of the FvMAPKs derived from four different programs.

| **Name** | **WoLF PSORT**^a^ | **CELLO**^b^ | **Plant-PLoc**^c^ | **ProtComp 9.0**^d^ |
| --- | --- | --- | --- | --- |
| *FvMAPK1* | nucl: 6, cyto: 5, chlo: 1, mito: 1 | Nuclear | Cytoplasm | Nuclear |
| *FvMAPK3* | nucl: 6, cyto: 6, cysk: 2 | Cytoplasmic | Cytoplasm | Nuclear |
| *FvMAPK4*-1 | cyto: 8, cysk: 4, nucl: 1 | Cytoplasmic | Chloroplast | Nuclear |
| *FvMAPK4*-2 | cyto: 10, mito: 2, nucl: 1 | Nuclear, Cytoplasmic, Mitochondrial | Cytoplasm | Nuclear |
| *FvMAPK6* | cysk: 5, nucl: 4, cyto: 4 | Cytoplasmic | Cytoplasm | Nuclear |
| *FvMAPK7* | nucl: 6, cyto: 4, mito: 2, plas: 1.5, golg_plas: 1.5 | Nuclear | Chloroplast | Nuclear |
| *FvMAPK9* | chlo: 7, nucl: 3, mito: 3 | Nuclear | Chloroplast | Cytoplasmic |
| *FvMAPK13* | extr: 4, chlo: 3, vacu: 3, cyto: 1, mito: 1, plas: 1 | Nuclear, Cytoplasmic | Cytoplasm | Nuclear |
| *FvMAPK16* | cyto: 8, nucl: 2, pero: 2, mito: 1 | Cytoplasmic, Nuclear | Mitochondrion | Nuclear |
| *FvMAPK17* | chlo: 8, nucl: 4, mito: 2 | Nuclear | Chloroplast | Nuclear |
| *FvMAPK19* | cyto: 11, nucl: 1, mito: 1 | Mitochondrial, Nuclear, Cytoplasmic | Chloroplast | Cytoplasmic |
| *FvMAPK20* | cyto: 6, pero: 3, nucl: 2, mito: 2 | Nuclear | Chloroplast | Cytoplasmic |

^a^ Localization prediction by WoLF PSORT (http://www.genscript.com/wolf-psort.html).

^b^ Localization prediction by CELLO v2.5 (http://cello.life.nctu.edu.tw/).

^c^ Localization prediction by Plant-PLoc (http://www.csbio.sjtu.edu.cn/bioinf/plant/).

^d^ Localization prediction by ProtComp 9.0 (http://linux1.softberry.com/berry.phtml?topic=protcomppl&group=programs&subgroup=proloc).

Abbreviations: nucl: nucleus, cyto: cytoplasm, chlo: chloroplast, mito: mitochondria, cysk: cytoskeleton, plas: plasma membrane, golg_plas: golgi apparatus and plasma membrane, extr: extracellular, vacu: vacuolar membrane, pero: peroxisome.
